# Health-related exit from employment before and during the COVID-19 pandemic in Norway: Analysis of population-wide register data 2013–2021

**DOI:** 10.1016/j.ssmph.2023.101598

**Published:** 2024-01-02

**Authors:** Kristian Heggebø, Jon Ivar Elstad

**Affiliations:** NOVA, OsloMet – Oslo Metropolitan University, P.O. Box 4, St. Olavs Plass, N-0130, Oslo, Norway

**Keywords:** Health-related labor market mobility, Economic conditions, Nordic welfare state, Health selection, Income maintenance schemes, Norway

## Abstract

People with health problems experience various labor market disadvantages, such as hiring discrimination and heightened risk of firing, but the impact of deteriorating economic conditions on health-related labor market mobility remains poorly understood. The strength of the downturn/crisis will most likely make a difference. During minor downturns, when few employees are made redundant, health-related exit may occur frequently since employers prefer to keep those with good health on the payroll. However, during major economic crises, when large-scale downsizing and firm closures abound, there will be less discretionary room for employers. Thus, some mechanisms that usually are damaging for people with health problems (e.g., seniority rules and negative connotations), can be neutralized, ultimately leading to smaller health differentials in labor market outcomes. The current study used population-wide administrative register data, covering the years 2013–2021, to examine health-related exit from employment (to unemployment/social assistance) before and during the COVID-19 pandemic in Norway. The pandemic spurred a major crisis on the Norwegian labor market and led to a record-high unemployment rate of 10.6 percent in March 2020. Restricting the analytical samples to labor market insiders, linear probability models showed that previous recipients of health-related benefits had a higher unemployment likelihood in the pre-crisis year 2019. The relative importance of poor health changed non-negligibly, however, during the COVID-19 pandemic. When identical statistical analyses were run on the crisis year 2020, health-related exit from employment was dampened. Yet, this labor market equalization was not followed by smaller health differentials in work income in 2021, mainly because people with good health retained or regained decent-paying jobs when the economic conditions improved again. In conclusion, major economic crises can lead to an equalization of labor market disadvantages for people with health problems, but health-related inequalities may reemerge when the economy recovers.

## Highlights

• Health-related employment exit was dampened during the 2020 COVID-19 pandemic.
• The income gap between people with good and poor health grew larger during 2021.
• Economic crises can lead to temporary reduction of health-related employment exit.
• Health-related labor market inequalities may reemerge when the economy recovers.

## 1 Introduction

Economic crises have repeatedly occurred in modern economies. The Great Depression, the 1973–74 oil crisis, and the 1990s dot-com bubble are well-known examples from the 20th century. More recent crises include the Great Recession, where the unemployment rate among European Union (EU) countries peaked at roughly 12 percent of the workforce in 2013 (Eurostat, 2023a), and the COVID-19 pandemic, which led to a 10.3 percent drop in gross domestic product (GDP) in the OECD area in the second quarter of 2020 (OECD, 2023a).

Typically, economic crises go together with adverse conditions for large parts of the population, such as unemployment, poverty, and marginalization. Economic downturns will often increase existing socioeconomic inequalities since disadvantages tend to be concentrated among low-status social groups (Heggebø et al., 2019). However, economic crises - and other major disruptions such as wars, plagues, and revolutions - can also lead to reduced inequalities if the playing field between “the haves” and “the have-nots” are levelled (Scheidel, 2018). Such effects may occur if the crises are deep enough to democratize the accompanying disadvantages. Thus, economic crises may not always lead to widening socioeconomic inequalities.

This paper addresses such topics by examining health-related exit from employment before and during the COVID-19 pandemic in Norway. The Norwegian labor market went suddenly from boom to bust, following the announcement of infection control measures on March 12, 2020. The unemployment rate rose immediately and soon reached a record-high level of 10.6 percent. Previous research has recurrently shown that people with health problems face labor market disadvantages (see e.g., Bjørnshagen, 2021; Butterworth et al., 2012; Riphahn, 1999). Their employment difficulties may be amplified when the economy takes a turn for the worse (Bartley & Owen, 1996), for instance if people with poor health tend to be fired first during downsizing processes. People with poor health have a higher likelihood of losing their jobs (see e.g., García-Gómez, Jones and Rice, 2010; Mastekaasa, 1996), and their employment rates have been observed to decline during minor economic downturns (see e.g., Minton, Pickett and Dorling, 2012; van der Wel, Dahl and Birkelund, 2010). It can nevertheless be suggested that sometimes, economic crises will lead to an equalization of labor market disadvantages (Heggebø & Dahl, 2015). This may occur during major crises characterized by mass unemployment which affect large sections of the working population and not in particular the lower-status occupations. Accordingly, we ask the following overarching research question: *Did the rapid deterioration of economic conditions during the COVID-19 pandemic in Norway lead to a strengthening, or a dampening, of health-related exit from employment?*

## 2 Previous research

### 2.1 Health-related labor market mobility

*Health-related labor market mobility* will usually refer to how entries into and exits from employment vary with people's health conditions. Those with poor health are overrepresented among the non-employed (Kaspersen et al., 2016) for three main reasons. First, people with health problems have lower job finding rates due to obstacles during recruitment processes such as hiring discrimination and employers' worry over high sickness absence (Bjørnshagen, 2021; Bjørnshagen & Ugreninov, 2021; García-Gómez, Jones and Rice, 2010). Second, people with poor health have a higher firing risk (Mastekaasa, 1996; Riphahn, 1999), partly due to lower seniority since the “last-in-first-out” principle prevails in many labor market segments. Third, individuals with deteriorated health tend to experience longer unemployment spells while out of work (Butterworth et al., 2012; Stewart, 2001), in part because of above-mentioned recruitment difficulties. They may therefore accumulate “unemployment scars” detrimental for future labor market prospects (Birkelund, Heggebø & Rogstad, 2017; Gangl, 2006). The labor market difficulties experienced by people with poor health exemplify *cumulative disadvantages* (DiPrete & Eirich, 2006; Leopold, 2016; Merton, 1968), where initial differences in resources (labor market attachment) between social groups (people with good vs. bad health) grow larger over time.

The health-employment relationship is reciprocal (Elstad, 1995; Korpi, 2001). People with poor health face labor market disadvantages because of their health conditions, and their health status may deteriorate further due to hardships experienced while out of work, as shown by studies of health consequences of unemployment (see e.g., Cooper, McCausland and Theodossiou, 2006; Eliason & Storrie, 2009; Iversen et al., 1987; Turner, 1995). Previous research also indicates that certain social groups (e.g., low educated, immigrants) are more susceptible to health-related exit from the labor market (Arrow, 1996; McDonough and Amick, 2001).

### 2.2 Economic conditions, health, and employment

Some previous research has addressed the impact of deteriorating macro-economic conditions on the association between health and employment. A survey-based study from the UK showed that men in manual occupations with limiting longstanding illness experienced more labor market disadvantages when economic conditions worsened during the 1980s (Bartley & Owen, 1996). A survey-based study from Norway found a similar pattern with increasing educational inequalities in employment among men and women with limiting longstanding illness during the 1988–1992 economic downturn in Norway, and few signs of decreasing inequalities when the economy recovered (van der Wel, Dahl & Birkelund, 2010).

Minton, Pickett and Dorling (2012) replicated the study by Bartley and Owen (1996). They covered a longer observational period (1973–2009) and found that the health-related employment gap had grown substantially over time in the UK, among both men and women. A survey-based study from Sweden showed that there was a particularly large decline in the employment rate of people with poor self-reported health during the 1990s crisis (Burström et al., 2012). Similarly, a study analyzing survey data from Canada, Denmark, Norway, Sweden, and the United Kingdom concluded that “… periods of high unemployment have sparked a downward trend in employment for already marginalized groups”, defined as people with short education and chronic health conditions (Holland et al., 2011).

All the above-mentioned studies are survey-based. The strengths of self-reported data, such as providing important information on social surroundings and subjective health status, are well-known, but there are also potential drawbacks, for instance non-random participation, drop-out, recall bias, and social desirability bias. Another commonality is that they only use employment as the outcome measure, suggesting that studies of other outcomes may shed broader light on health-related labor market mobility during economic crises. In addition, the reviewed literature has mostly examined minor economic downturns, but not major labor market crises. To our knowledge, few published papers have analyzed health-related exit from employment during the COVID-19 pandemic. However, two recent survey-based studies have analyzed outcomes that may be correlated with employment exit. In Canada, Bierman et al. (2021) showed that psychological distress and worse self-rated health were associated with more economic hardship during April–June 2020. In Norway, Bakkeli (2021) found that people with poor health more often reported worsened working conditions during the COVID-19 pandemic.

## 3 Theory, mechanisms and hypotheses

### 3.1 The association between poor health and employment

The causes (e.g., overproduction, oil embargo, bank collapse, housing bubbles, etc.) of economic crises differ, but they tend to have one thing in common: high unemployment. Numerous undesirable consequences are associated with the unemployment experience, as highlighted by Bartley (1994: 336): “losing a job can precipitate a self perpetuating series of negative events well into the future, even after work has been regained.” Examples of negative events include household income drop (Ehlert, 2012), poverty (Gallie, Paugam and Jacobs, 2003), material deprivation (Julkunen, 2002), separation (Gonalons-Pons & Gangl, 2021), weak future labor market attachment (Birkelund, Heggebø and Rogstad, 2017; Gangl, 2006), health deterioration (Cooper et al., 2006; Eliason and Storrie, 2009), and excess mortality (Gerdtham & Johannesson, 2003; Iversen et al., 1987).

Associations between poor health and unemployment and job loss are evident also when the economy is in booming and non-crisis phases. There are at least four reasons why employers prefer people with good health on the payroll. First, individuals with poor health have higher *sickness absence* and are thus more costly (e.g., need for temporary replacements). Second, poor health may – both explicitly and subconsciously – give employers *negative connotations* (i.e., personal characteristics correlated with worsened job performance). Third, people with poor health may experience *further health deterioration*, possibly to the point that they must leave the firm, which implies resource-consuming future recruitment processes. Fourth, last-in-first-out *seniority rules* will often play a role. People with poor health tend to have less seniority due to lower hiring likelihood, higher firing risk, and longer unemployment spells. Dismissal decisions based on seniority will therefore often lead to an overrepresentation of people with health problems.

### 3.2 Strength of the economic downturn/crisis

Economic crises differ as to how many become unemployed (i.e., the depth of the crisis) and as to how long the crisis lasts (i.e., the duration). Such circumstances will separate major crises from minor downturns, and this distinction may be of crucial importance. *Changes* in health-related labor market mobility during economic downturns and crises may provide some insight into the mechanisms involved (Hedström & Ylikoski, 2010).

Bartley (1988: 63) has argued that “a greater understanding of the economic issues of labour supply and demand … is necessary in order to advance further in understanding the interrelationship between unemployment and health”. The four above-mentioned mechanisms leading to employers’ preference for healthy employees have in common that employers often are risk averse while making decisions, but prevailing economic conditions are also likely to affect the weight given to health status during dismissal decisions. Whether labor demand is high or low is of obvious importance, but there may also be nuances along the continuum of labor demand. In other words, the *strength* of the downturn/crisis, and especially the number of individuals exposed (i.e., the depth), will probably matter.

During a slump, employers must decide which employees to keep, and which to let go. How small or large share of the workforce that is judged to be redundant will affect these decisions. If the entire firm is closed, dismissals may be non-selective – everyone is fired. Similarly, health status may not matter much when the main bulk of workers, for instance more than two-thirds, are to be fired. Large-scale downsizing processes give employers more limited room for maneuvering. Thus, during deep labor market crises, when firm closures and large-scale downsizing abound, the impact of health on employment exit may be dampened.

The discretionary room on the opposite side of the downsizing spectrum may also be limited. When only a few positions are redundant, the employees holding these specific positions will normally be let go, whatever their health. Exceptions may occur, however. Employers may reshuffle the workforce to keep the healthy employees that hold the redundant positions. The size of the firm/enterprise can also matter. In larger firms, multiple employees will perform the same work tasks, and employers will have to select which of the positions (and thus employees) that should be let go. Employers may therefore choose to fire an employee with health problems, for instance because of worries over sickness absence costs. Thus, during economic boom and milder downturns, when few employees lose their jobs, people with poor health are likely to be picked first.

Furthermore, the impact of health on labor market outcomes may vary between social groups, and it is conceivable that these interactions play out differently depending on the economic conditions. Individuals with immigrant background and/or short education could e.g., be especially prone to employment exit if health status is poor, at least when the economy is booming. Yet, such “double” labor market penalties might not appear during major economic crisis, when non-employment becomes much more widespread.

### 3.3 Hypotheses

Previous research has shown, first, that people with poor health are more prone to job loss, and second, that employment rates tend to decrease for people with health problems during mild economic downturns. There are nevertheless theoretical reasons why health should play a less important role for dismissal decisions when employers have to fire many employees simultaneously.

We therefore expect that health-related employment exit will occur frequently when rather few employees are made redundant, for instance during economic booms and minor downturns. The impact of health on employment exits may diminish, however, during major economic crises characterized by firm closures, mass layoffs, and large-scale downsizing.

Accordingly, the present study attempts to test the following two hypotheses.

Hypothesis 1 People with health problems have a higher likelihood of employment exit than people with good health during booming economic conditions.

Hypothesis 2 Health-related exit from employment is dampened during an economic crisis.

These hypotheses are investigated with Norwegian data – a country where a booming economy during the pre-COVID-19 years was followed by a steep increase in unemployment when the COVID-19 pandemic took hold during the Spring 2020.

## 4 Research context

### 4.1 The Norwegian welfare state

Norway is, in the typology of Esping-Andersen (1990), a social democratic welfare regime. Education is provided free-of-charge and/or heavily subsidized (via student stipends and loans with favorable interest rates) (OECD, 2020), and there are few out-of-pocket expenditures in the public healthcare system (EOHSP, 2022). Lengthy maternity and paternity leave are available (European Commission, 2023), and high kindergarten coverage eases the return to work for parents (OECD, 2015). Work-life balance has been a prioritized policy area, and the average Norwegian employee does not work unduly long hours (Eurofound, 2009). However, productivity at work tends to be rather high (NOU 2021: 2: p. 107–108). Furthermore, the Norwegian labor market, low-paying segments included, is characterized by high requirements for educational attainment and other credentials (e.g., course certifications). The prevalence of temporary work contracts is quite low (Eurostat, 2023b), and employment protection is relatively strong (OECD, 2023b; OECD, 2023c).

The Norwegian welfare state provides income support for those who are unable to make ends meet via income earned on the open labor market (NOU 2019: 7: table 5.1). The income maintenance schemes are mostly universal as opposed to means-tested (with one exception: social assistance). All inhabitants in Norway may apply for income support, of which different types exist (unemployment benefits, social assistance, sick pay, etc.), but there are strict requirements that need to be fulfilled before applications are granted.

### 4.2 From boom to bust

The first confirmed COVID-19 case was registered in Norway February 26, 2020. Two weeks later, on March 12, 2020 – the same day as the first confirmed COVID-19-related death in Norway – the Government introduced several infection control measures, including closure of bars, hairdressers, gyms, restaurants, etc. (NOU 2021: 6). Schools and kindergartens were closed during most of Spring 2020. Fear of SARS-CoV-2 contagion led to a slowdown of activity also in labor market segments not directly affected by the imposed infection control measures. The COVID-19 pandemic was comparatively mild in Norway during 2020, as measured by case rates, hospitalizations, and COVID-19-associated deaths (NOU 2021: 6: pp. 45–47). Large-scale rollout of vaccinations started in January 2021.

**Fig. 1:**
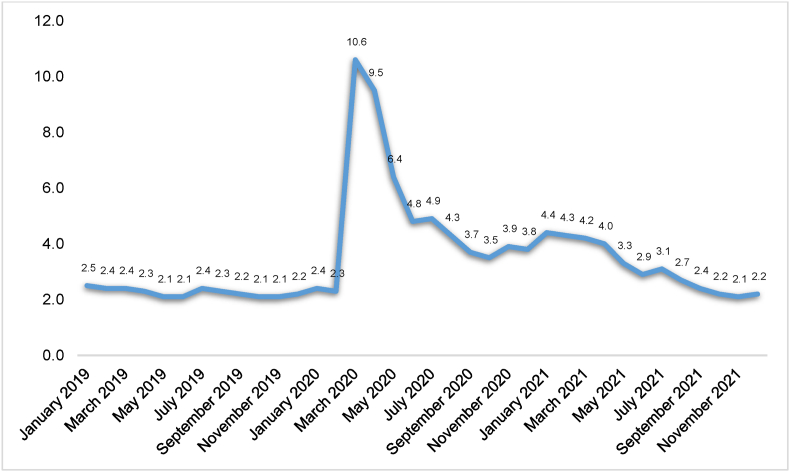
Registered monthly unemployment rate in Norway, January 2019–December 2021.

Source: NLWA (2023).

As evident from Fig. 1, the registered unemployment rate was very low, between 2.1 and 2.5, throughout 2019. The unemployment rate increased rapidly from 2.3 percent in February 2020 to 10.6 percent in March 2020 – a record-high registered monthly unemployment rate in Norway. The unemployment rate was more than four times higher in March–April 2020 than in January–February 2020. The unemployment rate stayed high during Spring 2020, before decreasing noticeably from June and onwards. Still, the unemployment rate was more than twice as high in June and July 2020 compared with the previous year. During 2021, the unemployment rate decreased slowly but steadily and reached pre-crisis levels in September. Thus, the crisis was deep at its peak but rather short-lived.

The Norwegian Government responded to the crisis by introducing a series of temporary amendments to the unemployment benefit legislation (NOU 2021: 6: 363–367), aimed at filling pre-existing gaps. As a result, more employees were covered by the unemployment benefit scheme in 2020 than in the non-crisis year 2019. Similar amendments were not introduced for other income maintenance schemes. The brunt of COVID-19-related unemployment episodes were temporary layoffs where the employment contract was paused rather than fully terminated (Heggebø & Pedersen, 2023). The unemployment prevalence was similar among workers with permanent contracts, temporary contracts, and the self-employed during Spring 2020 (Ingelsrud, 2021).

In summary, there are theoretical reasons to expect that health should be less consequential for dismissal decisions when employers must fire a large share of the workforce at the same time. Accordingly, the current study examines whether the 2020 labor market crisis in Norway was followed by less health-related exit from employment, compared with the pre-crisis situation in 2019. Next, we proceed with a description of data and methods.

## 5 Materials and methods

### 5.1 Data material

This paper analyzes administrative register data via the online analysis platform *microdata.no,* developed by Statistics Norway (SSB) and the Norwegian Agency for Shared Services in Education and Research (Sikt). The analysis platform holds longitudinal individual-level information for all registered inhabitants in Norway on e.g., sociodemographic information, social benefits, and various income sources. Researchers affiliated with certified Norwegian research institutions can access the data free of charge.

The register data consists of information that is collected routinely by various public authorities, for instance the Tax Administration, the Labor and Welfare Administration, and the Educational Loan Fund. Data are not collected for research purposes but have been made available for researchers by the respective data owners that use the information for administrative purposes (e.g., granting of social benefits or student loans). The data quality is enhanced by linking numerous administrative registries, holding information on e.g., educational attainment, work income, and household composition.

The individual-level information available in *microdata.no* has been linked and de-identified by SSB/Sikt via unique personal identification numbers, in accordance with Norwegian privacy legislation. Consequently, neither ethics approval nor informed consent is necessary as the analyzed data material is anonymized. The analysis platform has inbuilt security devices to ensure that users cannot access information about individual subjects.

### 5.2 Sample inclusion criteria

The analytical samples consist of individuals in prime working age, 30–62 years. People below 30 years are often still in education, whereas exit from the labor market (to e.g., retirement or disability benefit) becomes increasingly common above the age of 62.

We constructed two analytical samples consisting of *labor market insiders*, one sample for the pre-crisis year 2019 and another for the crisis year 2020. Labor market insiders were defined as individuals who earned minimum 3.5 times the *base amount* (BA) in work income during the years before 2019 or 2020, i.e., at least once during 2016–18 or 2017–19, respectively. The base amount (BA) is a sum of money, decided yearly by the Norwegian Parliament and used in the welfare system. BA reflects both price and wage changes and is therefore a well-suited measurement unit for income comparisons across years.

One BA was 99 858 Norwegian kroner (NOK) in 2019 and 101 351 NOK in 2020, i.e., roughly 10 000 Euros. The sum of 3.5 BA corresponds roughly to the yearly earnings of employees in the lowest-paying income brackets in Norway. We will also check whether the empirical results differ when the threshold for ‘labor market insider’ is set higher (i.e., earning 3.5 BA in two/three out of three years). Disability benefit recipients, people who died, and those who emigrated, were excluded from the two analytical samples.

### 5.3 Operationalization

People that received any unemployment benefits, i.e., more than zero Norwegian krone (NOK), in 2019/2020 were coded 1 on the outcome measure *unemployment* (else = 0). Job loss without finding new work will usually lead to receipt of unemployment benefits, especially for labor market insiders. Thus, code 1 on the outcome variable *unemployment* indicates exit from employment in 2016–18 (pre-crisis) or 2017–2019 (crisis) to unemployment for at least part of 2019/2020.

However, people with very low work income, as well as types of self-employed occupations, will not be eligible for unemployment benefits. When losing employment, they can apply for means-tested social assistance (Heggebø & Pedersen, 2023). Thus, social assistance receipt among labor market insiders may also indicate exit from employment. In supplementary analyses reported in the online material, social assistance is therefore used as an alternative outcome variable. Receipt of social assistance (more than zero NOK) in 2019/2020 was coded 1 on the variable *social assistance* (else = 0).

Receipt of two temporary health-related benefits – *work assessment allowance* and *sick pay* – is used for indicating health problems during some years before 2019 and 2020, i.e., as potential explanations for exit from employment. In order to be eligible for work assessment allowance, work capacity must be reduced by minimum 50 percent due to sickness or injury. Sickness absence needs to be certified by a physician in order to be granted sick pay. Those who received more than zero NOK in work assessment allowance in one or several of the years 2013–15/2014–16 were coded 1 (else = 0). Individuals who received more than 1 BA of sick pay in one or several years during 2013–15/2014–16 were coded 1 (else = 0) on the sick pay variable. Sickness absence less than 16 days is not covered by the sick pay scheme, and the criterion of minimum 1 BA was applied to exclude sickness absence of short-to-medium length (e.g., spells of 1–2 months).

Several sociodemographic variables are used as covariates. *Age* in years and *age squared* are included to take age effects and potential curvilinear relationship into account. Dummy variables for *woman* (men = 0) and being *married* (not married = 0) are included. Two dummy variables for *short education* (International Standard Classification of Education (ISCED) level = 0–3, 9) and *medium education* (ISCED level = 4–5) are included, with *long education* (ISCED level = 6–8) as the reference category. Finally, dummy variables for *immigrants* (born abroad = 1, else = 0), and *descendants* (born in Norway with immigrant parents = 1, else = 0) are included in the regressions. The covariates are measured in 2018 for the pre-crisis sample, and 2019 for the crisis sample.

### 5.4 Statistical methods

This study uses longitudinal register data to study health-related exit from employment. Analyses are run on two samples consisting of people with a history of health problems (indicated by receipt of health-related benefits), and healthy controls (i.e., individuals that have not received benefits due to health reasons). People who had poor health during a three-year period (2013–15/2014-16), and thereafter were labor market insiders in the subsequent three-year period (2016–18/2017-19), are followed up in two exposure years - pre-crisis (2019) and crisis (2020) - to examine risk of unemployment, compared to people with good health. Identical analyses are run for a second outcome measure: social assistance (results reported in supplementary online material, Tables S1, S2, and S4).

Data are analyzed with descriptive statistics and regressions. First, we describe the samples, split by exposure year (i.e., 2019 or 2020) and health (i.e., recipients of work assessment, recipients of sick pay, or healthy controls).

Thereafter, we estimate three linear probability models (i.e., ordinary least squares regressions [OLS]) with unemployment in 2019 as the outcome. Linear probability models are preferred since the estimated coefficients can be compared between varying models and samples in a straightforward manner. Comparing empirical findings between models and samples is more challenging in e.g., logistic regression because the coefficients can be affected by the degree of unobserved heterogeneity in the model specification (Allison, 1999; Mood, 2010). However, we will also estimate logistic regression models to see whether similar empirical findings appear. An additional advantage of linear probability models is that the derived coefficients are easy to interpret, i.e., as percentage point differences.

In Model 1, only the health variables are included as explanatory variables, i.e., dummy variables for work assessment allowance and sick pay receipt. The coefficients for these two explanatory variables indicate how the probability of unemployment in 2019 among labor market insiders differed between the healthy controls (the reference category) and those with health problems, indicated by receiving work assessment allowance, or sick pay, during 2013–2015. Model 2 adjusts for sociodemographic covariates (i.e., age and its square, woman, married, two education dummies, immigrant, and descendant). Model 3 adds 8 interaction terms between the two health indicators and education/immigrant background. Thereafter, identical linear probability (OLS) regression models are estimated for unemployment in 2020, with indicators of health problems during 2014–16 as the explanatory variables.

Comparing the coefficients for the two health indicators when analyzed for the pre-crisis 2019 sample and the crisis 2020 sample will shed light on the two hypotheses. However, coefficients obtained in multiple regression analyses may be misleading if the included independent variables correlate strongly. Such multi-collinearity problems are probably not a major problem here, as indicated by the correlation matrixes shown in supplementary material D (Tables S6 and S7). The strongest Pearson correlation coefficients (r) were for woman-long education (r = 0.204–0.209) and married-age (r = 0.175–0.181). The correlation between the two health indicators was also rather weak (r = 0.173–0.180). This suggests that it is unproblematic to include both explanatory variables simultaneously in regression models. However, we will also estimate statistical models with the explanatory variables included separately to see whether the results change.

After these regression analyses, several sub-group analyses and additional models are estimated. First, we estimate the main model with logistic regression, checking whether this alternative regression technique gives results similar to those obtained by the linear probability (OLS) regression approach. Second, a higher threshold is set for labor market insider, i.e., minimum two out of three years with more than 3.5 BA in work income during 2016–18/2017–19. Third, we exclude short-term unemployment, proxied by receipt of less than the median amount of unemployment benefits during 2019/2020. Fourth, long-term unemployment, defined as receiving more than 1BA in unemployment benefits during 2019/2020, is excluded. Fifth, only more serious health impairments, defined as receiving any work assessment allowance or more than one BA sick pay for minimum two years during 2013–15/2014–16, are included in the explanatory variable. Sixth, the results are run separately for men and women to examine potential gendered consequences of health on employment exit.

Finally, we examine whether the 2020 crisis was followed by any noticeable changes in health-related income inequalities. We do so by calculating average work income earned in the year before (2019) and after (2021) the crisis among people with health problems, as a percentage of what the healthy controls earned on average in the same observational years. This way, we can examine whether the health differentials in work income have decreased, stagnated, or increased in the aftermath of the COVID-19 pandemic.

## 6 Results

### 6.1 Descriptive statistics

Table 1 shows descriptive statistics, splitting the samples according to health status and exposure year (2019 and 2020). In both analytical samples, there are approximately 1.5 million healthy controls, roughly 180 000 previous sick pay recipients, and nearly 50 000 previous recipients of work assessment allowance.

**Table 1:** Descriptive statistics, split by health status and exposure year. Percent.

	Work assessment allowance		Sick pay (>1 BA)		Healthy controls	
	2019	2020	2019	2020	2019	2020
Unemployment	5.0 (N = 2.424)	16.2 (N = 7.508)	3.6 (N = 6.501)	13.5 (N = 24.482)	2.8 (N = 41.674)	14.1 (N = 213.926)
Soc. assistance	2.8 (N = 1.327)	2.6 (N = 1.219)	1.1 (N = 2.019)	1.1 (N = 1.945)	0.4 (N = 6.527)	0.5 (N = 7.001)
Age (in years)	45.5	45.5	46.4	46.5	45.4	45.3
Woman	54.7 (N = 26.360)	54.5 (N = 25.223)	57.7 (N = 105.282)	57.7 (N = 104.898)	42.6 (N = 637.243)	42.7 (N = 650.707)
Married	39.9 (N = 19.261)	38.6 (N = 17.872)	48.5 (N = 88.500)	48.0 (N = 87.121)	52.2 (N = 779.892)	51.4 (N = 781.755)
Education						
*Long*	36.5 (N = 17.591)	36.7 (N = 17.001)	40.0 (N = 72.944)	40.5 (N = 73.664)	47.2 (N = 705.982)	47.8 (N = 728.083)
*Medium*	36.7 (N = 17.696)	36.9 (N = 17.082)	37.3 (N = 68.045)	37.7 (N = 68.432)	34.5 (N = 515.341)	34.3 (N = 521.553)
*Short*	26.8 (N = 12.951)	26.4 (N = 12.216)	22.7 (N = 41.440)	21.8 (N = 39.606)	18.3 (N = 274.087)	17.9 (N = 272.667)
Immigrant	12.5 (N = 6.030)	13.0 (N = 6.019)	13.0 (N = 23.762)	13.8 (N = 25.048)	17.4 (N = 260.662)	18.3 (N = 278.361)
Descendant	0.6 (N = 298)	0.7 (N = 335)	0.7 (N = 1.208)	0.8 (N = 1.352)	0.5 (N = 7.983)	0.6 (N = 9.093)
N	48.233	46.289	182.429	181.703	1.495.413	1.522.316

**Notes:** Sample inclusion criteria: those who have earned minimum 3,5 times the base amount (BA) at least once during 2016–2018/2017–2019.

Poor health is measured as either (a) receipt of any work assessment allowance or (b) receipt of more than one base amount (BA) of sick pay, during 2013–2015/2014–2016.

Age span: 30–62 years.

Disability benefit recipients in 2019 and 2020 are excluded.

People who die or emigrate during the observational period are excluded.

The deteriorating economic conditions from 2019 to 2020 are clearly visible, with the unemployment share increasing more than threefold for recipients of both work assessment allowance (from 5.0 to 16.2 percent) and sick pay (from 3.6 to 13.5 percent). For the healthy controls, unemployment rose even more (from 2.8 percent in 2019 to 14.1 percent in 2020). There was, on the other hand, no increase as to social assistance receipt.

The healthy controls were more often male and married than people with indications of health problems, and they more often had long education. Short education was more prevalent among previous recipients of work assessment allowance than previous recipients of sick pay, and even less prevalent among the healthy controls. When compared to healthy controls, immigrants were underrepresented among the recipients of both work assessment allowance and sick pay, whereas descendants were slightly overrepresented.

### 6.2 Regression results

Table 2 shows results from linear probability (OLS) regression models of unemployment in 2019 (pre-crisis year) and 2020 (crisis year). Indicators of previous health problems during 2013–15/2014–16 are the explanatory variables of main interest. The coefficients for these two variables show how the probability of being unemployed in 2019 and 2020, respectively, *differed* from the unemployment probability among the healthy controls, i.e., the reference category.

**Table 2:** The impact of poor health on unemployment likelihood in pre-crisis (2019) and crisis (2020) years.

	Unemployment 2019			Unemployment 2020		
	Model 1	Model 2	Model 3	Model 1	Model 2	Model 3
Work assessment allowance	0.020* (0.001)	0.018* (0.001)	0.021* (0.001)	0.025* (0.002)	0.015* (0.002)	0.026* (0.003)
Sick pay	0.005* (0.000)	0.006* (0.000)	0.006* (0.001)	−0.009* (0.001)	−0.009* (0.001)	−0.003 (0.001)
Short education		0.026* (0.000)	0.027* (0.000)		0.117* (0.001)	0.119* (0.001)
Medium education		0.010* (0.000)	0.010* (0.000)		0.082* (0.001)	0.082* (0.001)
Immigrant		0.037* (0.000)	0.037* (0.000)		0.090* (0.001)	0.092* (0.001)
Descendant		0.016* (0.002)	0.014* (0.002)		0.040* (0.003)	0.040* (0.004)
Sociodemographic controls?	No	Yes	Yes	No	Yes	Yes
Interaction terms?	No	No	Yes	No	No	Yes
N		1.705.813			1.730.067	

**Notes:** Standard errors reported in parentheses.

* = coefficient significant on the 95 percent level.

Analysis method: Linear probability (ordinary least squares) regression models.

Outcome measure: Unemployment during 2019/2020.

Explanatory variables: Receipt of work assessment allowance (any) or sick pay (more than 1BA) during 2013–15/2014–16.

Sample inclusion criteria: labor market insiders, i.e., individuals earning more than 3.5 BA at least once during 2016–18/2017–19.

Age span: 30–62 years.

Model 1 includes the two health indicators only (i.e., work assessment allowance and sick pay). Healthy controls = reference category.

Model 2 adjusts for the following sociodemographic covariates: Short/medium education (ref: higher education), immigrant, descendant, age, age squared, gender (ref: males), and married (only coefficients for education and immigrant background are shown in the table).

Model 3 includes eight interaction terms between work assessment allowance/sick pay and short education, medium education, immigrant, and descendant.

Full models, with coefficients for all variables shown, are available in the online supplementary material B.

Before the COVID-19 pandemic, both health indicators were associated with a significantly higher unemployment likelihood than among the healthy controls. The coefficient size was considerably larger for work assessment allowance than for sick pay, with 2.0 and 0.5 percentage points higher probability of unemployment, respectively (model 1). Adding sociodemographic covariates (model 2) and then interaction terms (model 3) did not lead to noteworthy changes in these coefficients for how previous health problems affected risk of unemployment in 2019. Moreover, neither people with short/medium education nor individuals with immigrant background appear to be systematically more prone to health-related exit from employment (see Tables S3 and S4, supplementary material B for full models with interaction terms). The differences between the three statistical models appear to be small overall. Adjusting for sociodemographic covariates seems sensible while analyzing labor market outcomes, but the addition of 8 interaction terms makes the model overly complex. Thus, we will only comment on coefficients derived from the more parsimonious model 2 in the following.

**Fig. 2:**
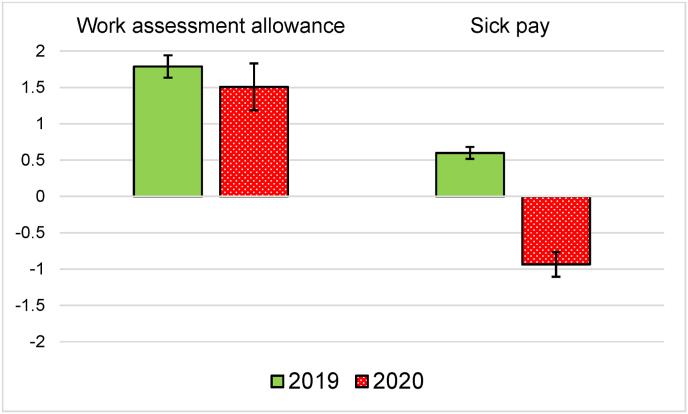
The impact of poor health on unemployment likelihood. Comparison between pre-crisis (2019) and crisis (2020) years. **Notes:** Results from linear probability (OLS) regression models. Coefficients derived from model 2 in Table 2. 95 percent confidence intervals included.

On analyzing unemployment in 2020 when the labor market was affected by the COVID-19 pandemic, the findings indicate that sick pay recipients had a significantly *lower* unemployment likelihood than the healthy controls – the coefficient is minus 0.9 percentage points (model 2). The coefficient size is rather small, but there is still a noticeable change of one and a half percentage points compared with 2019 (0.006 vs. −0.009). For work assessment allowance, the coefficient was quite similar in the two exposure years (0.018 vs. 0.015). These developments from the pre-crisis year 2019 to the crisis year 2020 are illustrated in Fig. 2. The coefficients are similar when the two explanatory variables, work assessment allowance (0.020 in 2019, 0.012 in 2020) and sick pay (0.008 in 2019, -0.008 in 2020), are included separately in the linear regression models, which indicates that multi-collinearity is not a major problem.

There was no increase in social assistance recipiency from 2019 to 2020 (cf. Table 1). Thus, there should not be noticeable changes over time in health-related exit from employment to social assistance. This is exactly what the empirical results show (Table S1, supplementary material A). The coefficients are nearly identical while comparing 2019 and 2020, across all three models. In model 2, social assistance likelihood is 1.8 (pre-crisis) and 2.0 (crisis) percentage points higher for work assessment allowance, and 0.4 percentage points higher for sick pay in both exposure years.

### 6.3 Sub-group analyses and additional models

Table 3 shows the results from sub-group analyses and additional models. The model set-up is identical as before, except that only models 1 and 2 are reported since the addition of interactions terms (model 3) did not alter the results much.

**Table 3:** The impact of poor health on unemployment likelihood in pre-crisis (2019) and crisis (2020) years. Results from various sub-group analyses and additional models.

	Unemployment 2019		Unemployment 2020	
	Model 1	Model 2	Model 1	Model 2
Sociodemographic controls?	No	Yes	No	Yes
**Panel A. Logistic regression model±**				
Work assessment allowance	1.70* (1.63, 1.77)	1.60* (1.53, 1.67)	1.22* (1.19, 1.25)	1.12* (1.10, 1.15)
Sick pay	1.19* (1.16, 1.22)	1.23* (1.19, 1.26)	0.93* (0.92, 0.94)	0.92* (0.91, 0.94)
N	1.705.813		1.730.067	
**Panel B. Firmer labor market attachment**				
Work assessment allowance	0.014* (0.001)	0.013* (0.001)	0.017* (0.002)	0.009* (0.002)
Sick pay	0.004* (0.000)	0.005* (0.000)	−0.008* (0.001)	−0.009* (0.001)
N	1.582.617		1.604.409	
**Panel C. Excluding short-term unemployment**				
Work assessment allowance	0.012* (0.001)	0.011* (0.001)	0.018* (0.001)	0.013* (0.001)
Sick pay	0.004* (0.000)	0.005* (0.000)	−0.002* (0.001)	−0.001 (0.001)
N	1.705.813		1.730.067	
**Panel D. Excluding long-term unemployment**				
Work assessment allowance	0.011* (0.001)	0.010* (0.001)	0.012* (0.001)	0.004* (0.001)
Sick pay	0.002* (0.000)	0.002* (0.000)	−0.011* (0.001)	−0.012* (0.001)
N	1.705.813		1.730.067	
**Panel E. More serious health impairment**				
Work assessment allowance	0.025* (0.001)	0.022* (0.001)	0.029* (0.002)	0.018* (0.002)
Sick pay	0.007* (0.001)	0.007* (0.001)	−0.011* (0.002)	−0.014* (0.002)
N	1.705.813		1.730.067	
**Panel F. Gender split Men**				
Work assessment allowance	0.028* (0.001)	0.024* (0.001)	0.039* (0.003)	0.025* (0.003)
Sick pay	0.012* (0.001)	0.010* (0.001)	0.014* (0.001)	0.004* (0.001)
N	948.167		960.511	
**Women**				
Work assessment allowance	0.014* (0.001)	0.013* (0.001)	0.016* (0.002)	0.007* (0.002)
Sick pay	0.002* (0.001)	0.002* (0.001)	−0.018* (0.001)	−0.020* (0.001)
N	757.639		769.564	

**Notes:** Standard errors reported in parentheses.

* = coefficient significant on the 95 percent level.

± = odds ratios and 95 percent confidence intervals (in parentheses) reported.

Analysis method: Logistic regression analysis in panel A, linear probability (ordinary least squares) regression models in panels B–F.

Outcome measure: Unemployment during 2019/2020.

Explanatory variables: Receipt of work assessment allowance (any) or sick pay (more than 1BA) during 2013–15/2014–16.

Sample inclusion criteria: labor market insiders, i.e., individuals earning more than 3.5 BA at least once during 2016–18/2017–19.

Age span: 30–62 years.

Firmer labor market attachment = minimum 2/3 years with >3.5 BA in work income during 2016–18/2017–19.

Short-term unemployment = less than the median in unemployment benefits (61 308 and 41 025 NOK in 2019 and 2020, respectively).

Long-term unemployment = more than 1BA in unemployment benefits.

More serious health impairment = receives any work assessment allowance or more than one BA sick pay for minimum 2/3 years during 2013–15/2014–16.

Model 1 includes the two health indicators only (i.e., work assessment allowance and sick pay). Healthy controls = reference category.

Model 2 adjusts for the following sociodemographic control variables: age, age squared, gender (ref.: males), married, two education dummies (ref.: higher education), immigrant, and descendant.

Results from logistic regression are shown in panel A. The odds ratio coefficient for work assessment allowance decreased from 1.60 in 2019 (pre-crisis) to 1.12 in 2020 (crisis), implying that the unemployment risk for recipients of work assessment allowance, relative to the corresponding risk among healthy controls, was significantly reduced from pre-crisis to the crisis year 2020. As to recipients of sick pay, the logistic regression coefficient was 0.92 in 2020, implying that in the crisis year, unemployment risk among the healthy controls was higher than among the previous recipients of sick pay.

The results are similar when the analytical sample was restricted to people with firmer labor market attachment, i.e., at least 3.5 BA in work income for two of the three years (panel B). Findings were also similar when the labor market insider definition was further narrowed and required minimum 3.5 BA in work income for all three years (results not shown). Excluding short-term unemployment (panel C) and long-term unemployment (panel D) from the models did not substantively alter the results. More serious health impairments are included in panel E, and coefficients are yet again quite similar to results shown in Table 2.

Panel F presents the results split by gender. Health-related exit from employment was more pronounced among men than women. It was especially among females that sick pay receipt is associated with lower unemployment likelihood during the COVID-19 pandemic.

Similar sub-group analyses have been estimated for social assistance as well (Table S2, supplementary material A). These analyses show, first, that health-related exit to social assistance was present for both health indicators, but, second, that effects of health problems on becoming social assistance recipients did not change significantly from 2019 to 2020.

### 6.4 Income differentials and labor market outcomes 2021

**Fig. 3:**
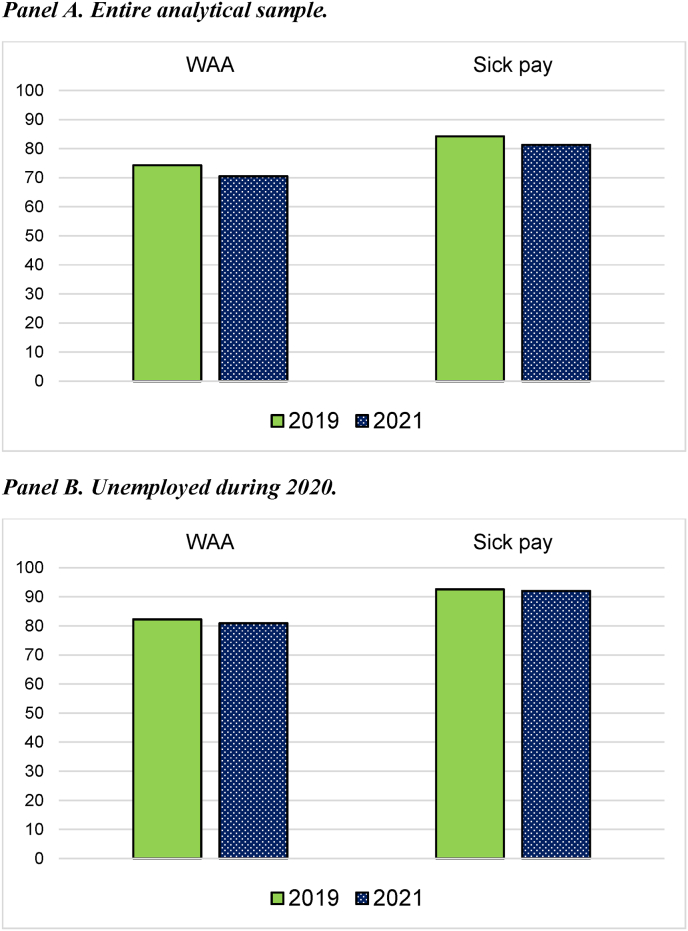
Health differentials in work income. Comparison between pre-crisis (2019) and post-crisis (2021) years. **Notes:** Differences in work income between people with poor health (i.e., previous recipients of work assessment allowance and sick pay) and healthy controls in pre-crisis (2019) and post-crisis (2021) years. The shares are computed by dividing the average work incomes among previous recipients of work assessment allowance/sick pay in 2019 and 2021 by the average work incomes among healthy controls in the same observational years. Panel A includes the entire analytical sample exposed to the 2020 crisis, whereas only those who experienced unemployment in 2020 are included in panel B.

Overall, findings indicate that health-related exit to unemployment was dampened, at least partly, in the crisis year 2020. This equalization of labor market disadvantages could, if sustained, also lead to smaller health differentials in income over time. By calculating average work income earned in the year before the crisis (2019) and after (2021), among people with and without health problems, Fig. 3 sheds light on this. Inclusion criteria are the same as before, except that only the second analytical sample is analyzed, i.e., those exposed to the 2020 crisis.

Previous recipients of work assessment allowance earned 74.26 percent of what the healthy controls did in 2019, but this share dropped to 70.48 percent in 2021 (panel A). Increasing health differentials in work income is observed for sick pay recipients as well (from 84.20 to 81.25 percent). When identical calculations are performed for individuals who experienced unemployment in 2020 (panel B), there was also a slight increase from 2019 to 2021 in health-related income differentials.

**Fig. 4:**
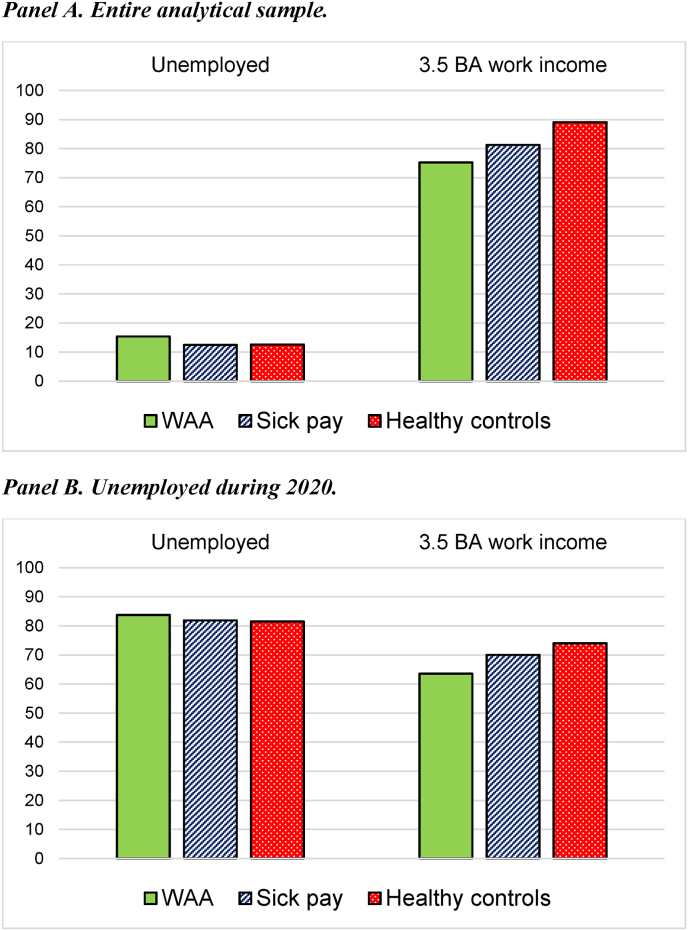
Health differentials in unemployment and firm labor market attachment during 2021. **Notes:** The figure shows how large proportion of previous recipients of work assessment allowance/sick pay and healthy controls that (i) received any unemployment benefits and/or (ii) earned more than 3.5 times the base amount (BA) in work income during the calendar year of 2021. Panel A includes the entire analytical sample exposed to the 2020 crisis, whereas only those who experienced unemployment in 2020 are included in panel B.

Some reasons why health-related income differentials have grown is suggested in Fig. 4. First, the healthy controls were unemployed less often, and second, earned work income equivalent to full-time employment more often in 2021, compared to people with poor health. This pattern was again similar for both the entire analytical sample (panel A) and for people who experienced unemployment during 2020 (panel B).

To summarize, less differences between those with health problems and the healthy controls in unemployment during the crisis year 2020, compared to the pre-crisis year 2019, was not followed by income equalization in 2021. On the contrary, differentials in work incomes according to health status appear to have increased somewhat in Norway in the aftermath of the COVID-19 pandemic.

## 7 Discussion

### 7.1 Summarizing discussion

Economic crises will probably arise anew, and knowledge about effects of deteriorating macro-economic conditions is needed. The current study contributes to this by examining health-related exit from employment before and during the COVID-19 pandemic in Norway, using population-wide administrative register data. Our overarching research question was: *Did the rapid deterioration of economic conditions during the COVID-19 pandemic in Norway lead to a strengthening, or a dampening, of health-related exit from employment?* This question was concretized into two hypotheses – (1) that people with health problems have a higher likelihood of employment exit than people with good health during booming economic conditions, while (2) health-related exit from employment is dampened during an economic crisis.

The analyses indicate that risk of exit from employment varied significantly with health conditions before the COVID-19 pandemic. Previous recipients of work assessment allowance – a benefit granted to people with minimum 50 percent reduced work capacity due to sickness or injury – who had returned to the labor market had roughly two percentage points higher unemployment likelihood in 2019 than healthy controls. The coefficient for work assessment allowance was almost on par with the labor market disadvantages experienced by people with short education, and can thus be considered to be of moderate size. Also individuals that previously had received sick pay for an extended period, had a heightened risk of unemployment, compared to people that could be assumed to be in good health. Thus, the *first hypothesis*, that people with poor health have a higher likelihood of employment exit during booming economic conditions, was supported.

The Norwegian labor market went from boom to bust shortly following the announcement of infection control measures in March 2020. When comparing 2019 and 2020, previous sick pay recipients went from having significantly higher to significantly lower unemployment risk, compared to people in good health, but there was no similar change when health status was indicated by previous receipt of work assessment allowance. These results were obtained by linear probability (OLS) regression models. When the models were estimated by logistic regression, effects on unemployment risk of both indicators of previous health problems, relative to the risk among healthy controls, were reduced noticeably in the 2020 crisis year, compared to the pre-crisis year 2019. Thus, overall, the *second hypothesis*, that health-related exit from employment is dampened during a major economic crisis, gained at least some support.

The existing literature on health and labor market attachment in changing economic conditions has indicated that employment rates tend to decline for people with health problems during milder economic downturns. The current paper extends previous research by indicating that health problems were less consequential for employment exit during the sudden major crisis on the Norwegian labor market caused by the COVID-19 pandemic.

Thus, this study lends some support to the idea that major economic crises can be an ‘equalizer’ (Scheidel, 2018) for people with health problems. There are theoretical reasons why major economic crisis can change the role of health status for employment and unemployment chances. During deep labor market crises, when large-scale downsizing and firm closures abound, there is less discretionary room for employers' dismissal decisions. Consequently, some explanatory mechanisms that usually contribute to exit from employment among people with poor health (e.g., seniority rules, sickness absence, negative connotations) can be weakened or neutralized, leading to smaller health differentials on the labor market.

The present study indicates that health-related exit from employment was somewhat dampened during the 2020 crisis year, but it is also noteworthy that this equalization was not followed by smaller health differentials in work income in 2021. The work income gap associated with health rather seemed to increase from 2019 to 2021. Thus, health-related labor market disadvantages, which might be reduced during a period of high unemployment, may reemerge as soon as the economic conditions start improving. In Norway, during September–December 2021, the unemployment rate returned to the pre-crisis levels, and in this booming period, people with good health retained and regained decent-paying jobs more often.

Accordingly, the tendencies to labor market equalization experienced by people with poor health during the COVID-19 pandemic in Norway turned out to be short-lived. Health-related inequalities tend to reemerge because of the mechanisms associated with employers interest in minimizing risk(s) when recruiting and promoting employees. As long as individuals with health problems are viewed as the less secure choice, health-related labor market inequalities will arise and endure. Policymakers, together with employers both in the private and public sector, should be encouraged to take steps to mute these mechanisms.

### 7.2 Strengths and limitations

The current study has used administrative register data 2013–2021, covering all registered inhabitants in Norway. In some respects, this is advantageous, compared to previous research which has relied on survey data in which issues such as non-random participation, attrition, and recall bias are more salient. A further strength of the present study is its focus on exit from employment among labor market insiders, which broadens the approach to these issues since earlier research has more often addressed employment rates.

There are, on the other hand, several limitations in the current paper. Its measures of health are proxies since they consist of previous receipt of health-related benefits. The assumption that *previous* receipt of health-related benefits is an indicator of *current* deteriorated health might not hold. Analyses of e.g., hospital admissions could have yielded different findings. Unobserved time trends, such as improved public health or increasing social inequalities in health during 2019–2021, can also introduce some bias.

The register data analyzed here have no self-assessed evaluations of health or other subjective information. Moreover, the registers do not cover undocumented migrants or other groups that are not included in Norwegian administrative registers. The current study did not have access to firm level information. Comparing downsizing (of various severity) and firm/plant closures may reveal important insights, and this could be an interesting avenue for future research. In-depth examinations - using both qualitative and quantitative methodology - of labor market segments hit particularly hard by the COVID-19 pandemic may also be a promising research topic.

Temporary changes of the unemployment insurance system during the COVID-19 pandemic (NOU 2021: 6: 363–367) could also lead to some bias. Table S5 (supplementary material C) does not show any clear compositional differences in sociodemographic variables between unemployment benefit recipients in 2019 and 2020, but the composition may still differ on unobservable characteristics (e.g., work motivation, personality, and cognitive abilities). In addition, with more people on the fringes of the labor market (Virtanen et al., 2003) covered by the scheme, it seems likely that more individuals with serious health conditions can apply for and receive unemployment benefits. The findings may therefore be upwardly biased in the 2020 crisis year, implying that health-related employment exit would have diminished even more without the temporary legislative changes. However, we do not know to what extent there are health differentials in the inclination to apply for unemployment benefits. There could also be a health component in the assessment and granting of unemployment benefit applications. Social workers may e.g., advice people with serious health conditions to rather apply for health-related benefits. These questions are left for future research.

Finally, the external validity of the current study may be limited due to peculiarities of the Norwegian labor market, characterized by e.g., rather high educational requirements and relatively strict labor market regulation.

### 7.3 Conclusion

The current study has examined health-related exit from employment before and during the COVID-19 pandemic in Norway, analyzing population-wide register data 2013–2021. Previous recipients of health-related benefits were more likely to experience exit to unemployment (and social assistance) in the pre-crisis year 2019. During the crisis year 2020, with unemployment peaking at 10.6 percent in March, there was a dampening of health-related employment exit. However, this labor market equalization was not followed by smaller health differentials in work income in 2021. On the contrary, the health-related income gap grew larger, mainly because those with good health more often retained or regained decent-paying jobs. Thus, major economic crises can lead to an equalization of labor market disadvantages for people with health problems, but health-related inequalities may reemerge as soon as the economic conditions improve again.

## Declaration of competing interest

The authors declare that we have no conflicts of interest.

## Funding

This work was supported by the 10.13039/501100005416Research Council of Norway [grant numbers 326136, 288638]. The funding body did not play any role in the study design, in the collection, analysis, and interpretation of the data, in the manuscript writing, or in the decision to submit the paper for publication.

## Ethical statement

The current research has been approved by the Norwegian Agency for Shared Services in Education and Research (Sikt) and by Statistics Norway (SSB). The individual-level register data information was linked and deidentified by SSB, in accordance with Norwegian privacy legislation. The ethics committees have waived the requirement to obtain informed consent as the register data analyzed in this study are in anonymized and deidentified format.

## CRediT authorship contribution statement

**Kristian Heggebø:** Conceptualization, Data curation, Formal analysis, Investigation, Methodology, Writing – original draft, Writing – review & editing. **Jon Ivar Elstad:** Conceptualization, Investigation, Methodology, Writing – review & editing, Validation.

## Data Availability

The data that support the findings of this study are available at the online analysis platform microdata.no. The syntax is included in the online supplementary material E.

## References

[bib59] Allison P.D. (1999). Comparing logit and probit coefficients across groups. Sociological Methods & Research.

[bib1] Arrow J.O. (1996). Estimating the influence of health as a risk factor on unemployment: A survival analysis of employment durations for workers surveyed in the German socio- economic panel (1984–1990). Social Science & Medicine.

[bib2] Bakkeli N.Z. (2021). Health, work, and contributing factors on life satisfaction: A study in Norway before and during the COVID-19 pandemic. SSM-Population Health.

[bib3] Bartley M. (1988). Unemployment and health: Selection or causation‐a false antithesis?. Sociology of Health & Illness.

[bib4] Bartley M. (1994). Unemployment and ill health: Understanding the relationship. Journal of Epidemiology & Community Health.

[bib5] Bartley M., Owen C. (1996). Relation between socioeconomic status, employment, and health during economic change, 1973-93. BMJ.

[bib6] Bierman A., Upenieks L., Glavin P., Schieman S. (2021). Accumulation of economic hardship and health during the COVID-19 pandemic: Social causation or selection?. Social Science & Medicine.

[bib7] Birkelund G.E., Heggebø K., Rogstad J. (2017). Additive or multiplicative disadvantage? The scarring effects of unemployment for ethnic minorities. European Sociological Review.

[bib8] Bjørnshagen V. (2021). The mark of mental health problems. A field experiment on hiring discrimination before and during COVID-19. Social Science & Medicine.

[bib9] Bjørnshagen V., Ugreninov E. (2021). Disability disadvantage: Experimental evidence of hiring discrimination against wheelchair users. European Sociological Review.

[bib10] Burström B., Nylén L., Barr B., Clayton S., Holland P., Whitehead M. (2012). Delayed and differential effects of the economic crisis in Sweden in the 1990s on health-related exclusion from the labour market: A health equity assessment. Social Science & Medicine.

[bib11] Butterworth P., Leach L.S., Pirkis J., Kelaher M. (2012). Poor mental health influences risk and duration of unemployment: A prospective study. Social Psychiatry and Psychiatric Epidemiology.

[bib12] Cooper D., McCausland W.D., Theodossiou I. (2006). The health hazards of unemployment and poor education: The socioeconomic determinants of health duration in the European union. Economics and Human Biology.

[bib13] DiPrete T.A., Eirich G.M. (2006). Cumulative advantage as a mechanism for inequality: A review of theoretical and empirical developments. Annual Review of Sociology.

[bib14] Ehlert M. (2012). Buffering income loss due to unemployment: Family and welfare state influences on income after job loss in the United States and western Germany. Social Science Research.

[bib15] Eliason M., Storrie D. (2009). Job loss is bad for your health–Swedish evidence on cause- specific hospitalization following involuntary job loss. Social Science & Medicine.

[bib16] Elstad J.I. (1995). Employment status and women's health - exploring the dynamics. Acta Sociologica.

[bib17] EOHSP (2022).

[bib18] Esping-Andersen G. (1990).

[bib19] Eurofound (2009). Working time in the European Union: Norway. https://www.eurofound.europa.eu/publications/report/2009/working-time-in-the-european-union-norway.

[bib20] European Commission (2023). Norway - benefits for pregnancy, birth and adoption. https://ec.europa.eu/social/main.jsp?catId=1123&intPageId=4704&langId=en.

[bib21] Eurostat (2023). Unemployment by sex and age - monthly data. https://ec.europa.eu/eurostat/databrowser/view/une_rt_m/default/table?lang=en.

[bib22] Eurostat (2023). Part-time employment and temporary contracts - annual data. https://ec.europa.eu/eurostat/databrowser/view/LFSI_PT_A__custom_4900112/default/table?lang=en.

[bib23] Gallie D., Paugam S., Jacobs S. (2003). Unemployment, poverty and social isolation: Is there a vicious circle of social exclusion?. European Societies.

[bib24] Gangl M. (2006). Scar effects of unemployment: An assessment of institutional complementarities. American Sociological Review.

[bib25] García-Gómez P., Jones A.M., Rice N. (2010). Health effects on labour market exits and entries. Labour Economics.

[bib26] Gerdtham U.G., Johannesson M. (2003). A note on the effect of unemployment on mortality. Journal of Health Economics.

[bib27] Gonalons-Pons P., Gangl M. (2021). Marriage and masculinity: Male-breadwinner culture, unemployment, and separation risk in 29 countries. American Sociological Review.

[bib28] Hedström P., Ylikoski P. (2010). Causal mechanisms in the social sciences. Annual Review of Sociology.

[bib29] Heggebø K., Dahl E. (2015). Unemployment and health selection in diverging economic conditions: Compositional changes? Evidence from 28 European countries. International Journal for Equity in Health.

[bib30] Heggebø K., Pedersen A.W., Takle M., Vedeler J.S., Schoyen M.A., Klette-Bøhler K., Falch-Eriksen A. (2023). Citizenship and social exclusion at the margins of the welfare state.

[bib31] Heggebø K., Tøge A.G., Dahl E., Berg J.E. (2019). Socioeconomic inequalities in health during the Great recession: A scoping review of the research literature. Scandinavian Journal of Public Health.

[bib32] Holland P., Burström B., Whitehead M., Diderichsen F., Dahl E., Barr B., Uppal S. (2011). How do macro-level contexts and policies affect the employment chances of chronically ill and disabled people? Part I: The impact of recession and deindustrialization. International Journal of Health Services.

[bib33] Ingelsrud M.H. (2021). Standard and non-standard working arrangements in Norway – consequences of COVID-19. Labour & Industry: A Journal of the Social and Economic Relations of Work.

[bib34] Iversen L., Andersen O., Andersen P.K., Christoffersen K., Keiding N. (1987). Unemployment and mortality in Denmark, 1970-80. British Medical Journal (Clinical research ed.).

[bib35] Julkunen I. (2002). Social and material deprivation among unemployed youth in Northern Europe. Social Policy and Administration.

[bib36] Kaspersen S.L., Pape K., Vie G.Å., Ose S.O., Krokstad S., Gunnell D., Bjørngaard J.H. (2016). Health and unemployment: 14 years of follow-up on job loss in the Norwegian HUNT study. The European Journal of Public Health.

[bib37] Korpi T. (2001). Accumulating disadvantage. Longitudinal analyses of unemployment and physical health in representative samples of the Swedish population. European Sociological Review.

[bib38] Leopold L. (2016). Cumulative advantage in an egalitarian country? Socioeconomic health disparities over the life course in Sweden. Journal of Health and Social Behavior.

[bib39] Mastekaasa A. (1996). Unemployment and health: Selection effects. Journal of Community & Applied Social Psychology.

[bib40] McDonough P., Amick B.C. (2001). The social context of health selection: A longitudinal study of health and employment. Social Science & Medicine.

[bib41] Merton R.K. (1968). The Matthew effect in science. Science.

[bib42] Minton J.W., Pickett K.E., Dorling D. (2012). Health, employment, and economic change, 1973-2009: Repeated cross sectional study. BMJ.

[bib58] Mood C. (2010). Logistic regression: Why we cannot do what we think we can do, and what we can do about it. European Sociological Review.

[bib43] NLWA (2023). *Antall helt ledige historisk. Juni 2023. Beholdning helt ledige som andel av arbeidsstyrken*. Norwegian Labor and Welfare Administration. https://www.nav.no/no/nav-og-samfunn/statistikk/arbeidssokere-og-stillinger-statistikk/hovedtall-om-arbeidsmarkedet.

[bib44] NOU 2019: 7 (2019). *Arbeid og inntektssikring - tiltak for økt sysselsetting*. Oslo: Ministry of Labour and Social Inclusion. https://www.regjeringen.no/no/dokumenter/nou-2019-7/id2637967/?ch=1.

[bib45] NOU 2021: 2 (2021). Kompetanse, aktivitet og inntektssikring — Tiltak for økt sysselsetting. Oslo: Ministry of Labour and Social Inclusion. https://www.regjeringen.no/contentassets/2943e48dbf4544b8b5f456c850dcccbe/no/pdfs/nou202120210002000dddpdfs.pdf.

[bib46] NOU 2021: 6 (2021). Myndighetenes håndtering av koronapandemien — Rapport fra Koronakommisjonen. Oslo: Office of the Prime Minister. https://www.regjeringen.no/no/dokumenter/nou-2021-6/id2844388/.

[bib47] OECD (2015). Early childhood Education and care policy review. Norway. https://www.oecd.org/norway/early-childhood-education-and-care-policy-review-norway.pdf.

[bib48] OECD (2020). Education policy outlook: Norway. https://www.oecd.org/education/policy-outlook/country-profile-Norway-2020.pdf.

[bib49] OECD (2023). Quarterly national accounts: Quarterly growth rates of real GDP. https://stats.oecd.org/index.aspx?queryid=350.

[bib50] OECD (2023). *Strictness of employment protection – individual and collective dismissals (regular contracts*). https://stats.oecd.org/Index.aspx?DataSetCode=EPL_OV.

[bib51] OECD (2023). Strictness of employment protection – temporary contracts. https://stats.oecd.org/Index.aspx?DataSetCode=EPL_T.

[bib52] Riphahn R.T. (1999). Income and employment effects of health shocks: A test case for the German welfare state. Journal of Population Economics.

[bib53] Scheidel W. (2018).

[bib54] Stewart J.M. (2001). The impact of health status on the duration of unemployment spells and the implications for studies of the impact of unemployment on health status. Journal of Health Economics.

[bib55] Turner J.B. (1995). Economic context and the health effects of unemployment. Journal of Health and Social Behavior.

[bib56] van der Wel K.A., Dahl E., Birkelund G.E. (2010). Employment inequalities through busts and booms: The changing roles of health and education in Norway 1980- 2005. Acta Sociologica.

[bib57] Virtanen P., Liukkonen V., Vahtera J., Kivimäki M., Koskenvuo M. (2003). Health inequalities in the workforce: The labour market core–periphery structure. International Journal of Epidemiology.

