# Supplementary material for: Health-related exit from employment before and during the COVID-19 pandemic in Norway: Analysis of population-wide register data 2013–2021
Source: SSM Popul Health. 2024 Jan 2;25:101598. doi: 10.1016/j.ssmph.2023.101598 (PMC10818249; doi:10.1016/j.ssmph.2023.101598)
Supplement: Multimedia component 1 [file mmc1.docx]

Health-related exit from employment before and during the COVID-19 pandemic in Norway: Analysis of population-wide administrative data 2013-2021

# Supplementary material A: Social assistance

**Table S1. The impact of poor health on social assistance likelihood in pre-crisis (2019) and crisis (2020) years.**

|  | | Social assistance 2019 | | | Social assistance 2020 | | |
| --- | --- | --- | --- | --- | --- | --- | --- |
|  | | Model 1 | Model 2 | Model 3 | Model 1 | Model 2 | Model 3 |
| Work assessment allowance | | 0.021* (0.001) | 0.020* (0.001) | 0.009* (0.001) | 0.020* (0.000) | 0.018* (0.000) | 0.008* (0.001) |
| Sick pay | | 0.004* (0.000) | 0.004* (0.000) | 0.002* (0.000) | 0.004* (0.000) | 0.004* (0.000) | 0.002* (0.000) |
| Short education | |  | 0.013* (0.000) | 0.012* (0.000) |  | 0.014* (0.000) | 0.013* (0.000) |
| Medium education | |  | 0.003* (0.000) | 0.003* (0.000) |  | 0.003* (0.000) | 0.003* (0.000) |
| Immigrant | |  | 0.008* (0.000) | 0.007* (0.000) |  | 0.008* (0.000) | 0.007* (0.000) |
| Descendant | |  | 0.001 (0.001) | 0.000 (0.001) |  | 0.002* (0.001) | 0.003* (0.001) |
| Sociodemographic controls? | | No | Yes | Yes | No | Yes | Yes |
| Interaction terms? | | No | No | Yes | No | No | Yes |
| N | | 1.705.908 | | | 1.730.177 | | |
| Notes | Standard errors reported in parentheses.  * = coefficient significant on the 95 percent level.  Analysis method: Linear probability (ordinary least squares) regression models.  Outcome measure: Social assistance during 2019/2020.  Explanatory variables: Receipt of work assessment allowance (any) or sick pay (more than 1BA) during 2013-15/2014-16.  Sample inclusion criteria: labor market insiders, i.e., individuals earning more than 3.5 BA at least once during 2016-18/2017-19.  Model 1 includes the two health indicators only (i.e., work assessment allowance and sick pay). Healthy controls = reference category.  Model 2 adjusts for the following sociodemographic covariates: Short/medium education (ref: higher education), immigrant, descendant, age, age squared, gender (ref: males), and married (only coefficients for education and immigrant background are shown in the table).  Model 3 includes eight interaction terms between work assessment allowance/sick pay and short education, medium education, immigrant, and descendant.  Full models, with coefficients for all variables shown, are available in the online supplementary material B. | | | | | | |

**Table S2. The impact of poor health on social assistance likelihood in pre-crisis (2019) and crisis (2020) years. Results from various sub-group analyses and additional models.**

|  | | Social assistance 2019 | | Social assistance 2020 | |
| --- | --- | --- | --- | --- | --- |
|  | | Model 1 | Model 2 | Model 1 | Model 2 |
| Sociodemographic controls? | | No | Yes | No | Yes |
| **Panel A. Logistic regression model±** | | | | | |
| Work assessment allowance | | 4.71* (4.43, 5.02) | 3.77* (3.54, 4.02) | 4.40* (4.12, 4.69) | 3.42* (3.20, 3.65) |
| Sick pay | | 1.75* (1.66, 1.84) | 1.80* (1.70, 1.89) | 1.65* (1.56, 1.74) | 1.71* (1.62, 1.80) |
| N | | 1.705.908 | | 1.730.177 | |
| **Panel B. Firmer labor market attachment** | | | | | |
| Work assessment allowance | | 0.013* (0.000) | 0.012* (0.000) | 0.013* (0.000) | 0.012* (0.000) |
| Sick pay | | 0.003* (0.000) | 0.003* (0.000) | 0.003* (0.000) | 0.003* (0.000) |
| N | | 1.582.692 | | 1.604.492 | |
| **Panel C. Excluding short-term social assistance receipt** | | | | | |
| Work assessment allowance | | 0.002* (0.000) | 0.001* (0.000) | 0.001* (0.000) | 0.001* (0.000) |
| Sick pay | | 0.000* (0.000) | 0.000* (0.000) | 0.000* (0.000) | 0.000* (0.000) |
| N | | 1.705.908 | | 1.730.177 | |
| **Panel D. Excluding long-term social assistance receipt** | | | | | |
| Work assessment allowance | | 0.020* (0.000) | 0.018* (0.000) | 0.019* (0.000) | 0.017* (0.000) |
| Sick pay | | 0.004* (0.000) | 0.004* (0.000) | 0.004* (0.000) | 0.003* (0.000) |
| N | | 1.705.908 | | 1.730.177 | |
| **Panel E. More serious health impairment** | | | | | |
| Work assessment allowance | | 0.025* (0.000) | 0.023* (0.000) | 0.024* (0.000) | 0.022* (0.000) |
| Sick pay | | 0.008* (0.000) | 0.008* (0.000) | 0.007* (0.000) | 0.007* (0.000) |
| N | | 1.705.908 | | 1.730.177 | |
| **Panel F. Gender split**  **Men** | | | | | |
| Work assessment allowance | | 0.031* (0.001) | 0.028* (0.001) | 0.028* (0.001) | 0.025* (0.001) |
| Sick pay | | 0.008* (0.000) | 0.006* (0.000) | 0.007* (0.000) | 0.005* (0.000) |
| N | | 948.256 | | 960.602 | |
| **Women** | | | | | |
| Work assessment allowance | | 0.013* (0.000) | 0.012* (0.000) | 0.013* (0.000) | 0.012* (0.000) |
| Sick pay | | 0.002* (0.000) | 0.002* (0.000) | 0.002* (0.000) | 0.002* (0.000) |
| N | | 757.652 | | 769.575 | |
| Notes | Standard errors reported in parentheses.  * = coefficient significant on the 95 percent level.  ± = odds ratios and 95 percent confidence intervals (in parentheses) reported.  Analysis method: Logistic regression analysis in panel A, linear probability (ordinary least squares) regression models in panels B-F.  Outcome measure: Social assistance during 2019/2020.  Explanatory variables: Receipt of work assessment allowance (any) or sick pay (more than 1BA) during 2013-15/2014-16.  Sample inclusion criteria: labor market insiders, i.e., individuals earning more than 3.5 BA at least once during 2016-18/2017-19.  Age span: 30-62 years.  Firmer labor market attachment = minimum 2/3 years with > 3.5 BA in work income during 2016-18/2017-19.  Short-term social assistance receipt = less than 1BA in social assistance.  Long-term social assistance receipt = more than 1BA in social assistance.  More serious health impairment = receives any work assessment allowance or more than one BA sick pay for minimum 2/3 years during 2013-15/2014-16.  Model 1 includes the two health indicators only (i.e., work assessment allowance and sick pay). Healthy controls = reference category.  Model 2 adjusts for the following sociodemographic control variables: age, age squared, gender (ref.: males), married, two education dummies (ref.: higher education), immigrant, and descendant. | | | | |

# Supplementary material B: Full models

***Table S3. The impact of poor health on unemployment likelihood in pre-crisis (2019) and crisis (2020) years. Full model, all coefficients shown.***

|  | | Unemployment 2019 | | | Unemployment 2020 | | |
| --- | --- | --- | --- | --- | --- | --- | --- |
|  | | Model 1 | Model 2 | Model 3 | Model 1 | Model 2 | Model 3 |
| Work assessment allowance | | 0.020* (0.001) | 0.018* (0.001) | 0.021* (0.001) | 0.025* (0.002) | 0.015* (0.002) | 0.026* (0.003) |
| Sick pay | | 0.005* (0.000) | 0.006* (0.000) | 0.006* (0.001) | -0.009* (0.001) | -0.009* (0.001) | -0.003 (0.001) |
| Age | |  | -0.001* (0.000) | -0.001* (0.000) |  | -0.002* (0.000) | -0.002* (0.000) |
| Age^2^ | |  | 0.000* (0.000) | 0.000* (0.000) |  | 0.000 (0.000) | 0.000 (0.000) |
| Woman | |  | -0.003* (0.000) | -0.003* (0.000) |  | -0.012* (0.001) | -0.012* (0.001) |
| Married | |  | -0.011* (0.000) | -0.011* (0.000) |  | -0.019* (0.001) | -0.019* (0.001) |
| Short education | |  | 0.026* (0.000) | 0.027* (0.000) |  | 0.117* (0.001) | 0.119* (0.001) |
| Medium education | |  | 0.010* (0.000) | 0.010* (0.000) |  | 0.082* (0.001) | 0.082* (0.001) |
| Immigrant | |  | 0.037* (0.000) | 0.037* (0.000) |  | 0.090* (0.001) | 0.092* (0.001) |
| Descendant | |  | 0.016* (0.002) | 0.014* (0.002) |  | 0.040* (0.003) | 0.040* (0.004) |
| Short education*work assessment allowance | |  |  | 0.002 (0.002) |  |  | -0.011* (0.004) |
| Short education*sick pay | |  |  | -0.004* (0.001) |  |  | -0.013* (0.002) |
| Medium education*work assessment allowance | |  |  | -0.004* (0.002) |  |  | -0.013* (0.004) |
| Medium education*sick pay | |  |  | 0.001 (0.001) |  |  | -0.006* (0.002) |
| Immigrant*work assessment allowance | |  |  | -0.014* (0.002) |  |  | -0.023* (0.005) |
| Immigrant*sick pay | |  |  | 0.002 (0.001) |  |  | -0.013* (0.002) |
| Descendant*work assessment allowance | |  |  | -0.023* (0.010) |  |  | -0.025 (0.019) |
| Descendant*sick pay | |  |  | 0.021* (0.005) |  |  | 0.007 (0.010) |
| Sociodemographic controls? | | No | Yes | Yes | No | Yes | Yes |
| Interaction terms? | | No | No | Yes | No | No | Yes |
| N | | 1.705.813 | | | 1.730.067 | | |
| Notes | Standard errors reported in parentheses.  * = coefficient significant on the 95 percent level.  Analysis method: Linear probability (ordinary least squares) regression models.  Outcome measure: Unemployment during 2019/2020.  Explanatory variables: Receipt of work assessment allowance (any) or sick pay (more than 1BA) during 2013-15/2014-16.  Sample inclusion criteria: labor market insiders, i.e., individuals earning more than 3.5 BA at least once during 2016-18/2017-19.  Age span: 30-62 years.  Model 1 includes the two health indicators only (i.e., work assessment allowance and sick pay). Healthy controls = reference category.  Model 2 adjusts for the following sociodemographic covariates: Short/medium education (ref: higher education), immigrant, descendant, age, age squared, gender (ref: males), and married (only coefficients for education and immigrant background are shown in the table).  Model 3 includes eight interaction terms between work assessment allowance/sick pay and short education, medium education, immigrant, and descendant. | | | | | | |

***Table S4. The impact of poor health on social assistance likelihood in pre-crisis (2019) and crisis (2020) years. Full model, all coefficients shown.***

|  | | Social assistance 2019 | | | Social assistance 2020 | | |
| --- | --- | --- | --- | --- | --- | --- | --- |
|  | | Model 1 | Model 2 | Model 3 | Model 1 | Model 2 | Model 3 |
| Work assessment allowance | | 0.021* (0.001) | 0.020* (0.001) | 0.009* (0.001) | 0.020* (0.000) | 0.018* (0.000) | 0.008* (0.001) |
| Sick pay | | 0.004* (0.000) | 0.004* (0.000) | 0.002* (0.000) | 0.004* (0.000) | 0.004* (0.000) | 0.002* (0.000) |
| Age | |  | 0.001* (0.000) | 0.001* (0.000) |  | 0.001* (0.000) | 0.001* (0.000) |
| Age^2^ | |  | -0.000* (0.000) | -0.000* (0.000) |  | -0.000* (0.000) | -0.000* (0.000) |
| Woman | |  | -0.002* (0.000) | -0.002* (0.000) |  | -0.002* (0.000) | -0.002* (0.000) |
| Married | |  | -0.006* (0.000) | -0.006* (0.000) |  | -0.006* (0.000) | -0.006* (0.000) |
| Short education | |  | 0.013* (0.000) | 0.012* (0.000) |  | 0.014* (0.000) | 0.013* (0.000) |
| Medium education | |  | 0.003* (0.000) | 0.003* (0.000) |  | 0.003* (0.000) | 0.003* (0.000) |
| Immigrant | |  | 0.008* (0.000) | 0.007* (0.000) |  | 0.008* (0.000) | 0.007* (0.000) |
| Descendant | |  | 0.001 (0.001) | 0.000 (0.001) |  | 0.002* (0.001) | 0.003* (0.001) |
| Short education*work assessment allowance | |  |  | 0.028* (0.001) |  |  | 0.028* (0.001) |
| Short education*sick pay | |  |  | 0.002* (0.000) |  |  | 0.002* (0.001) |
| Medium education*work assessment allowance | |  |  | 0.009* (0.001) |  |  | 0.009* (0.001) |
| Medium education*sick pay | |  |  | 0.002* (0.000) |  |  | 0.002* (0.000) |
| Immigrant*work assessment allowance | |  |  | 0.002* (0.001) |  |  | -0.005* (0.001) |
| Immigrant*sick pay | |  |  | 0.003* (0.001) |  |  | 0.004* (0.001) |
| Descendant*work assessment allowance | |  |  | -0.002 (0.004) |  |  | -0.006 (0.004) |
| Descendant*sick pay | |  |  | 0.004 (0.002) |  |  | -0.001 (0.002) |
| Sociodemographic controls? | | No | Yes | Yes | No | Yes | Yes |
| Interaction terms? | | No | No | Yes | No | No | Yes |
| N | | 1.705.908 | | | 1.730.177 | | |
| Notes | Standard errors reported in parentheses.  * = coefficient significant on the 95 percent level.  Analysis method: Linear probability (ordinary least squares) regression models.  Outcome measure: Social assistance during 2019/2020.  Explanatory variables: Receipt of work assessment allowance (any) or sick pay (more than 1BA) during 2013-15/2014-16.  Sample inclusion criteria: labor market insiders, i.e., individuals earning more than 3.5 BA at least once during 2016-18/2017-19.  Model 1 includes the two health indicators only (i.e., work assessment allowance and sick pay). Healthy controls = reference category.  Model 2 adjusts for the following sociodemographic covariates: Short/medium education (ref: higher education), immigrant, descendant, age, age squared, gender (ref: males), and married (only coefficients for education and immigrant background are shown in the table).  Model 3 includes eight interaction terms between work assessment allowance/sick pay and short education, medium education, immigrant, and descendant. | | | | | | |

# Supplementary material C: Compositional differences among unemployment benefit recipients, 2019 vs. 2020

**Table S5. Descriptive statistics among unemployment benefit recipients in pre-crisis (2019) and crisis (2020) years. Percent.**

|  | | 2019 | 2020 |
| --- | --- | --- | --- |
| Work assessment allowance | | 4.87 | 3.09 |
| Sick pay | | 13.06 | 10.08 |
| Age (in years) | | 43.75 | 44.21 |
| Woman | | 38.58 | 38.67 |
| Married | | 42.12 | 46.28 |
| Education | |  |  |
| *Long* | | 34.10 | 31.58 |
| *Medium* | | 35.86 | 42.29 |
| *Short* | | 27.61 | 23.61 |
| Immigrant | | 34.53 | 27.23 |
| Descendant | | 0.04 | 0.03 |
| N | | 49 814 | 242 941 |
| Notes | Sample inclusion criteria: those who have earned minimum 3,5 times the base amount (BA) at least once during 2016-2018/2017-2019.  Subsample consisting of unemployment benefit recipients during 2019/2020.  Poor health is measured as either (a) receipt of any work assessment allowance or (b) receipt of more than one base amount (BA) of sick pay, during 2013-2015/2014-2016.  Age span: 30-62 years.  Disability benefit recipients in 2019 and 2020 are excluded.  People who die or emigrate during the observational period are excluded. | | |

Supplementary material D: Correlation matrix

**Table S6. Correlation matrix for variables included in regression analysis, 2019 pre-crisis analytical sample.**

|  | **WAA**  **2013-15** | **Sick pay 2013-15** | **Age** | **Woman** | **Married** | **Long education** | **Medium education** | **Short education** | **Immigrant** | **Descendant** |
| --- | --- | --- | --- | --- | --- | --- | --- | --- | --- | --- |
| **Unemployed 2019** | 0.021 | 0.013 | -0.032 | -0.020 | -0.033 | -0.042 | 0.004 | 0.052 | 0.082 | 0.006 |
| **WAA**  **2013-15** | - | 0.173 | 0.001 | 0.035 | -0.040 | -0.034 | 0.007 | 0.044 | -0.020 | 0.002 |
| **Sick pay 2013-15** | 0.173 | - | 0.035 | 0.093 | -0.021 | -0.044 | 0.018 | 0.048 | -0.036 | 0.005 |
| **Age** | 0.001 | 0.035 | - | -0.002 | 0.175 | -0.110 | 0.033 | 0.140 | -0.143 | -0.062 |
| **Woman** | 0.035 | 0.093 | -0.002 | - | -0.003 | 0.204 | -0.137 | -0.068 | -0.056 | 0.001 |
| **Married** | -0.040 | -0.021 | 0.175 | -0.003 | - | 0.050 | -0.032 | -0.031 | 0.083 | 0.008 |
| **Long education** | -0.034 | -0.044 | -0.110 | 0.204 | 0.050 | - | -0.678 | -0.413 | -0.049 | 0.011 |
| **Medium education** | 0.007 | 0.018 | 0.033 | -0.137 | -0.032 | -0.678 | - | -0.325 | -0.079 | -0.011 |
| **Short education** | 0.044 | 0.048 | 0.140 | -0.068 | -0.031 | -0.413 | -0.325 | - | 0.029 | 0.003 |
| **Immigrant** | -0.020 | -0.036 | -0.143 | -0.056 | 0.083 | -0.049 | -0.079 | 0.029 | - | -0.034 |

**Table S7. Correlation matrix for variables included in regression analysis, 2020 crisis analytical sample.**

|  | **WAA**  **2014-16** | **Sick pay 2014-16** | **Age** | **Woman** | **Married** | **Long education** | **Medium education** | **Short education** | **Immigrant** | **Descendant** |
| --- | --- | --- | --- | --- | --- | --- | --- | --- | --- | --- |
| **Unemployed 2020** | 0.010 | -0.006 | -0.054 | -0.047 | -0.036 | -0.124 | 0.065 | 0.087 | 0.101 | 0.008 |
| **WAA**  **2014-16** | - | 0.180 | 0.001 | 0.033 | -0.040 | -0.034 | 0.008 | 0.045 | -0.021 | 0.003 |
| **Sick pay 2014-16** | 0.180 | - | 0.040 | 0.091 | -0.019 | -0.044 | 0.022 | 0.046 | -0.035 | 0.006 |
| **Age** | 0.001 | 0.040 | - | -0.003 | 0.181 | -0.109 | 0.045 | 0.128 | -0.138 | -0.067 |
| **Woman** | 0.033 | 0.091 | -0.003 | - | -0.001 | 0.209 | -0.137 | -0.073 | -0.055 | 0.001 |
| **Married** | -0.040 | -0.019 | 0.181 | -0.001 | - | 0.051 | -0.032 | -0.033 | 0.085 | 0.007 |
| **Long education** | -0.034 | -0.044 | -0.109 | 0.209 | 0.051 | - | -0.684 | -0.407 | -0.056 | 0.012 |
| **Medium education** | 0.008 | 0.022 | 0.045 | -0.137 | -0.032 | -0.684 | - | -0.315 | -0.084 | -0.012 |
| **Short education** | 0.045 | 0.046 | 0.128 | -0.073 | -0.033 | -0.407 | -0.315 | - | 0.036 | 0.003 |
| **Immigrant** | -0.021 | -0.035 | -0.138 | -0.055 | 0.085 | -0.056 | -0.084 | 0.036 | - | -0.037 |

# Supplementary material E: Syntax

// CONNECTING TO SSB DATA

require no.ssb.fdb:22 as db

// HEALTH-RELATED EXIT FROM EMPLOYMENT, COVID-19 PANDEMIC, NORWAY

// OUTCOME: UNEMPLOYMENT

// observational years: 2013-2021

create-dataset health_exit_COVID19

import db/BEFOLKNING_KJOENN as gender

import db/SIVSTANDFDT_SIVSTAND 2018-11-01 as marital_stat_2018

import db/SIVSTANDFDT_SIVSTAND 2019-11-01 as marital_stat_2019

import db/NUDB_BU 2018-11-01 as education_2018

import db/NUDB_BU 2019-11-01 as education_2019

import db/BEFOLKNING_FOEDSELS_AAR_MND as birth_year_month

import db/BEFOLKNING_INVKAT as immi_category

import db/BEFOLKNING_STATUSKODE 2013-01-01 as reg_stat_2013

import db/BEFOLKNING_STATUSKODE 2014-01-01 as reg_stat_2014

import db/BEFOLKNING_STATUSKODE 2015-01-01 as reg_stat_2015

import db/BEFOLKNING_STATUSKODE 2016-01-01 as reg_stat_2016

import db/BEFOLKNING_STATUSKODE 2017-01-01 as reg_stat_2017

import db/BEFOLKNING_STATUSKODE 2018-01-01 as reg_stat_2018

import db/BEFOLKNING_STATUSKODE 2019-01-01 as reg_stat_2019

import db/BEFOLKNING_STATUSKODE 2020-01-01 as reg_stat_2020

import db/BEFOLKNING_STATUSKODE 2021-01-01 as reg_stat_2021

import db/INNTEKT_WYRKINNT 2016-11-01 as work_income_2016

import db/INNTEKT_WYRKINNT 2017-11-01 as work_income_2017

import db/INNTEKT_WYRKINNT 2018-11-01 as work_income_2018

import db/INNTEKT_WYRKINNT 2019-11-01 as work_income_2019

import db/INNTEKT_WYRKINNT 2020-11-01 as work_income_2020

import db/INNTEKT_WYRKINNT 2021-11-01 as work_income_2021

import db/INNTEKT_SYKEPENGER 2013-11-01 as sick_pay_2013

import db/INNTEKT_SYKEPENGER 2014-11-01 as sick_pay_2014

import db/INNTEKT_SYKEPENGER 2015-11-01 as sick_pay_2015

import db/INNTEKT_SYKEPENGER 2016-11-01 as sick_pay_2016

import db/INNTEKT_SUM_ARBAVKL 2013-11-01 as WAA_2013

import db/INNTEKT_SUM_ARBAVKL 2014-11-01 as WAA_2014

import db/INNTEKT_SUM_ARBAVKL 2015-11-01 as WAA_2015

import db/INNTEKT_SUM_ARBAVKL 2016-11-01 as WAA_2016

import db/INNTEKT_ARBLED 2016-11-01 as unemp_ben_2016

import db/INNTEKT_ARBLED 2017-11-01 as unemp_ben_2017

import db/INNTEKT_ARBLED 2018-11-01 as unemp_ben_2018

import db/INNTEKT_ARBLED 2019-11-01 as unemp_ben_2019

import db/INNTEKT_ARBLED 2020-11-01 as unemp_ben_2020

import db/INNTEKT_ARBLED 2021-11-01 as unemp_ben_2021

import db/INNTEKT_KODE218 2019-11-01 as disab_ben_2019

import db/INNTEKT_KODE218 2020-11-01 as disab_ben_2020

replace work_income_2016 = 0 if sysmiss (work_income_2016)

replace work_income_2017 = 0 if sysmiss (work_income_2017)

replace work_income_2018 = 0 if sysmiss (work_income_2018)

replace work_income_2019 = 0 if sysmiss (work_income_2019)

replace work_income_2020 = 0 if sysmiss (work_income_2020)

replace work_income_2021 = 0 if sysmiss (work_income_2021)

replace sick_pay_2013 = 0 if sysmiss (sick_pay_2013)

replace sick_pay_2014 = 0 if sysmiss (sick_pay_2014)

replace sick_pay_2015 = 0 if sysmiss (sick_pay_2015)

replace sick_pay_2016 = 0 if sysmiss (sick_pay_2016)

replace WAA_2013 = 0 if sysmiss (WAA_2013)

replace WAA_2014 = 0 if sysmiss (WAA_2014)

replace WAA_2015 = 0 if sysmiss (WAA_2015)

replace WAA_2016 = 0 if sysmiss (WAA_2016)

replace unemp_ben_2016 = 0 if sysmiss (unemp_ben_2016)

replace unemp_ben_2017 = 0 if sysmiss (unemp_ben_2017)

replace unemp_ben_2018 = 0 if sysmiss (unemp_ben_2018)

replace unemp_ben_2019 = 0 if sysmiss (unemp_ben_2019)

replace unemp_ben_2020 = 0 if sysmiss (unemp_ben_2020)

replace unemp_ben_2021 = 0 if sysmiss (unemp_ben_2021)

replace disab_ben_2019 = 0 if sysmiss (disab_ben_2019)

replace disab_ben_2020 = 0 if sysmiss (disab_ben_2020)

// CODING COVARIATES, years: 2018-19

generate married_2018 = 0

replace married_2018 = 1 if marital_stat_2018 == '2'

generate married_2019 = 0

replace married_2019 = 1 if marital_stat_2019 == '2'

generate woman = 0

replace woman = 1 if gender == '2'

generate age_2018 = 2018 - int(birth_year_month/100)

generate age2_2018 = age_2018 * age_2018

generate age_2019 = 2019 - int(birth_year_month/100)

generate age2_2019 = age_2019 * age_2019

generate educ_level_2018 = substr(education_2018, 1,1)

generate educ_high_2018 = 0

replace educ_high_2018 = 1 if educ_level_2018 == '6' | educ_level_2018 == '7' | educ_level_2018 == '8'

generate educ_medium_2018 = 0

replace educ_medium_2018 = 1 if educ_level_2018 == '4' | educ_level_2018 == '5'

generate educ_low_2018 = 0

replace educ_low_2018 = 1 if educ_level_2018 == '3' | educ_level_2018 == '2' | educ_level_2018 == '1' | educ_level_2018 == '0' | educ_level_2018 == '9'

generate educ_level_2019 = substr(education_2019, 1,1)

generate educ_high_2019 = 0

replace educ_high_2019 = 1 if educ_level_2019 == '6' | educ_level_2019 == '7' | educ_level_2019 == '8'

generate educ_medium_2019 = 0

replace educ_medium_2019 = 1 if educ_level_2019 == '4' | educ_level_2019 == '5'

generate educ_low_2019 = 0

replace educ_low_2019 = 1 if educ_level_2019 == '3' | educ_level_2019 == '2' | educ_level_2019 == '1' | educ_level_2019 == '0' | educ_level_2019 == '9'

generate majo = 0

replace majo = 1 if immi_category == 'A'

generate immi = 0

replace immi = 1 if immi_category == 'B'

generate desc = 0

replace desc = 1 if immi_category == 'C'

drop marital_stat_2018 marital_stat_2019 gender education_2018 educ_level_2018 education_2019 educ_level_2019 immi_category

// TRIMMING SAMPLE: DROP YOUNG AND OLD

drop if age_2019 < 20

drop if age_2019 > 78

// CODING LM VARIABLES

generate unemp_2016 = 0

replace unemp_2016 = 1 if unemp_ben_2016 > 0

generate unemp_2017 = 0

replace unemp_2017 = 1 if unemp_ben_2017 > 0

generate unemp_2018 = 0

replace unemp_2018 = 1 if unemp_ben_2018 > 0

generate unemp_2019 = 0

replace unemp_2019 = 1 if unemp_ben_2019 > 0

generate unemp_2020 = 0

replace unemp_2020 = 1 if unemp_ben_2020 > 0

generate unemp_2021 = 0

replace unemp_2021 = 1 if unemp_ben_2021 > 0

generate BA_3_5_2016 = 0

replace BA_3_5_2016 = 1 if work_income_2016 >= 324016

generate BA_3_5_2017 = 0

replace BA_3_5_2017 = 1 if work_income_2017 >= 327719

generate BA_3_5_2018 = 0

replace BA_3_5_2018 = 1 if work_income_2018 >= 339091

generate BA_3_5_2019 = 0

replace BA_3_5_2019 = 1 if work_income_2019 >= 349503

generate BA_3_5_2020 = 0

replace BA_3_5_2020 = 1 if work_income_2020 >= 354729

generate BA_3_5_2021 = 0

replace BA_3_5_2021 = 1 if work_income_2021 >= 372397

generate firm_emp_2016_18 = 0

replace firm_emp_2016_18 = 1 if BA_3_5_2016 == 1 | BA_3_5_2017 == 1 | BA_3_5_2018 == 1

generate firm_emp_2017_19 = 0

replace firm_emp_2017_19 = 1 if BA_3_5_2017 == 1 | BA_3_5_2018 == 1 | BA_3_5_2019 == 1

generate firm_emp_min2Y_2016_18 = BA_3_5_2016 + BA_3_5_2017 + BA_3_5_2018

recode firm_emp_min2Y_2016_18 (0=0) (1=0) (2=1) (3=1)

generate firm_emp_min2Y_2017_19 = BA_3_5_2017 + BA_3_5_2018 + BA_3_5_2019

recode firm_emp_min2Y_2017_19 (0=0) (1=0) (2=1) (3=1)

generate unemp_max1BA_2019 = 0

replace unemp_max1BA_2019 = 1 if unemp_ben_2019 > 0 & unemp_ben_2019 < 99858

generate unemp_max1BA_2020 = 0

replace unemp_max1BA_2020 = 1 if unemp_ben_2020 > 0 & unemp_ben_2020 < 101351

generate unemp_max1BA_2021 = 0

replace unemp_max1BA_2021 = 1 if unemp_ben_2021 > 0 & unemp_ben_2020 < 106399

generate unemp_median_2019 = 0

replace unemp_median_2019 = 1 if unemp_ben_2019 >= 61300

generate unemp_median_2020 = 0

replace unemp_median_2020 = 1 if unemp_ben_2020 >= 42000

generate unemp_median_2021 = 0

replace unemp_median_2021 = 1 if unemp_ben_2021 >= 121000

// CODING HEALTH INDICATORS

generate sickpay_2013_dum = 0

replace sickpay_2013_dum = 1 if sick_pay_2013 > 0

generate sickpay_2014_dum = 0

replace sickpay_2014_dum = 1 if sick_pay_2014 > 0

generate sickpay_2015_dum = 0

replace sickpay_2015_dum = 1 if sick_pay_2015 > 0

generate sickpay_2016_dum = 0

replace sickpay_2016_dum = 1 if sick_pay_2016 > 0

generate sickpay_2013_15 = 0

replace sickpay_2013_15 = 1 if sickpay_2013_dum == 1 | sickpay_2014_dum == 1 | sickpay_2015_dum == 1

generate sickpay_2014_16 = 0

replace sickpay_2014_16 = 1 if sickpay_2014_dum == 1 | sickpay_2015_dum == 1 | sickpay_2016_dum == 1

generate sickpay_min2Y_2013_15 = sickpay_2013_dum + sickpay_2014_dum + sickpay_2015_dum

recode sickpay_min2Y_2013_15 (0=0) (1=0) (2=1) (3=1)

generate sickpay_min2Y_2014_16 = sickpay_2014_dum + sickpay_2015_dum + sickpay_2016_dum

recode sickpay_min2Y_2014_16 (0=0) (1=0) (2=1) (3=1)

generate sickpay_2013_1BA = 0

replace sickpay_2013_1BA = 1 if sick_pay_2013 >= 85245

generate sickpay_2014_1BA = 0

replace sickpay_2014_1BA = 1 if sick_pay_2014 >= 88370

generate sickpay_2015_1BA = 0

replace sickpay_2015_1BA = 1 if sick_pay_2015 >= 90068

generate sickpay_2016_1BA = 0

replace sickpay_2016_1BA = 1 if sick_pay_2016 >= 92576

generate sickpay_2013_15_1BA = 0

replace sickpay_2013_15_1BA = 1 if sickpay_2013_1BA == 1 | sickpay_2014_1BA == 1 | sickpay_2015_1BA == 1

generate sickpay_2014_16_1BA = 0

replace sickpay_2014_16_1BA = 1 if sickpay_2014_1BA == 1 | sickpay_2015_1BA == 1 | sickpay_2016_1BA == 1

generate sickpay_min2Y_2013_15_1BA = sickpay_2013_1BA + sickpay_2014_1BA + sickpay_2015_1BA

recode sickpay_min2Y_2013_15_1BA (0=0) (1=0) (2=1) (3=1)

generate sickpay_min2Y_2014_16_1BA = sickpay_2014_1BA + sickpay_2015_1BA + sickpay_2016_1BA

recode sickpay_min2Y_2014_16_1BA (0=0) (1=0) (2=1) (3=1)

generate WAA_2013_dum = 0

replace WAA_2013_dum = 1 if WAA_2013 > 0

generate WAA_2014_dum = 0

replace WAA_2014_dum = 1 if WAA_2014 > 0

generate WAA_2015_dum = 0

replace WAA_2015_dum = 1 if WAA_2015 > 0

generate WAA_2016_dum = 0

replace WAA_2016_dum = 1 if WAA_2016 > 0

generate WAA_2013_15 = 0

replace WAA_2013_15 = 1 if WAA_2013_dum == 1 | WAA_2014_dum == 1 | WAA_2015_dum == 1

generate WAA_2014_16 = 0

replace WAA_2014_16 = 1 if WAA_2014_dum == 1 | WAA_2015_dum == 1 | WAA_2016_dum == 1

generate WAA_min2Y_2013_15 = WAA_2013_dum + WAA_2014_dum + WAA_2015_dum

recode WAA_min2Y_2013_15 (0=0) (1=0) (2=1) (3=1)

generate WAA_min2Y_2014_16 = WAA_2014_dum + WAA_2015_dum + WAA_2016_dum

recode WAA_min2Y_2014_16 (0=0) (1=0) (2=1) (3=1)

generate poor_health_2013 = 0

replace poor_health_2013 = 1 if WAA_2013 > 0 | sick_pay_2013 > 0

generate poor_health_2014 = 0

replace poor_health_2014 = 1 if WAA_2014 > 0 | sick_pay_2014 > 0

generate poor_health_2015 = 0

replace poor_health_2015 = 1 if WAA_2015 > 0 | sick_pay_2015 > 0

generate poor_health_2016 = 0

replace poor_health_2016 = 1 if WAA_2016 > 0 | sick_pay_2016 > 0

generate poor_health_2013_15 = 0

replace poor_health_2013_15 = 1 if poor_health_2013 == 1 | poor_health_2014 == 1 | poor_health_2015 == 1

generate poor_health_2014_16 = 0

replace poor_health_2014_16 = 1 if poor_health_2014 == 1 | poor_health_2015 == 1 | poor_health_2016 == 1

generate poor_health_min2Y_2013_15 = poor_health_2013 + poor_health_2014 + poor_health_2015

recode poor_health_min2Y_2013_15 (0=0) (1=0) (2=1) (3=1)

generate poor_health_min2Y_2014_16 = poor_health_2014 + poor_health_2015 + poor_health_2016

recode poor_health_min2Y_2014_16 (0=0) (1=0) (2=1) (3=1)

generate disabled_2019 = 0

replace disabled_2019 = 1 if disab_ben_2019 > 0

generate disabled_2020 = 0

replace disabled_2020 = 1 if disab_ben_2020 > 0

drop sick_pay_2013 sick_pay_2014 sick_pay_2015 sick_pay_2016 WAA_2013 WAA_2014 WAA_2015 WAA_2016 unemp_ben_2016 unemp_ben_2017 unemp_ben_2018 unemp_ben_2019 unemp_ben_2020 disab_ben_2019 disab_ben_2020

generate WAA_immi_1315 = immi * WAA_2013_15

generate WAA_desc_1315 = desc * WAA_2013_15

generate WAA_low_edu_1315 = educ_low_2018 * WAA_2013_15

generate WAA_med_edu_1315 = educ_medium_2018 * WAA_2013_15

generate WAA_immi_1416 = immi * WAA_2014_16

generate WAA_desc_1416 = desc * WAA_2014_16

generate WAA_low_edu_1416 = educ_low_2019 * WAA_2014_16

generate WAA_med_edu_1416 = educ_medium_2019 * WAA_2014_16

generate sickp_immi_1315 = immi * sickpay_2013_15_1BA

generate sickp_desc_1315 = desc * sickpay_2013_15_1BA

generate sickp_low_edu_1315 = educ_low_2018 * sickpay_2013_15_1BA

generate sickp_med_edu_1315 = educ_medium_2018 * sickpay_2013_15_1BA

generate sickp_immi_1416 = immi * sickpay_2014_16_1BA

generate sickp_desc_1416 = desc * sickpay_2014_16_1BA

generate sickp_low_edu_1416 = educ_low_2019 * sickpay_2014_16_1BA

generate sickp_med_edu_1416 = educ_medium_2019 * sickpay_2014_16_1BA

// DESCRIPTIVE STATISTICS

// 2019

summarize unemp_2019 age_2018 woman married_2018 educ_high_2018 educ_medium_2018 educ_low_2018 immi desc if reg_stat_2019 == '1' & firm_emp_2016_18 == 1 & inrange(age_2018, 30, 62) & WAA_2013_15 == 1 & disabled_2019 == 0

summarize unemp_2019 age_2018 woman married_2018 educ_high_2018 educ_medium_2018 educ_low_2018 immi desc if reg_stat_2019 == '1' & firm_emp_2016_18 == 1 & inrange(age_2018, 30, 62) & sickpay_2013_15_1BA == 1 & disabled_2019 == 0

summarize unemp_2019 age_2018 woman married_2018 educ_high_2018 educ_medium_2018 educ_low_2018 immi desc if reg_stat_2019 == '1' & firm_emp_2016_18 == 1 & inrange(age_2018, 30, 62) & WAA_2013_15 == 0 & sickpay_2013_15_1BA == 0 & disabled_2019 == 0

// 2020

summarize unemp_2020 age_2019 woman married_2019 educ_high_2019 educ_medium_2019 educ_low_2019 immi desc if reg_stat_2020 == '1' & firm_emp_2017_19 == 1 & inrange(age_2019, 30, 62) & WAA_2014_16 == 1 & disabled_2020 == 0

summarize unemp_2020 age_2019 woman married_2019 educ_high_2019 educ_medium_2019 educ_low_2019 immi desc if reg_stat_2020 == '1' & firm_emp_2017_19 == 1 & inrange(age_2019, 30, 62) & sickpay_2014_16_1BA == 1 & disabled_2020 == 0

summarize unemp_2020 age_2019 woman married_2019 educ_high_2019 educ_medium_2019 educ_low_2019 immi desc if reg_stat_2020 == '1' & firm_emp_2017_19 == 1 & inrange(age_2019, 30, 62) & WAA_2014_16 == 0 & sickpay_2014_16_1BA == 0 & disabled_2020 == 0

// sum stats for descendants

// 2019

tabulate woman if desc == 1 & reg_stat_2019 == '1' & firm_emp_2016_18 == 1 & inrange(age_2018, 30, 62) & WAA_2013_15 == 1 & disabled_2019 == 0

tabulate woman if desc == 1 & reg_stat_2019 == '1' & firm_emp_2016_18 == 1 & inrange(age_2018, 30, 62) & sickpay_2013_15_1BA == 1 & disabled_2019 == 0

tabulate woman if desc == 1 & reg_stat_2019 == '1' & firm_emp_2016_18 == 1 & inrange(age_2018, 30, 62) & WAA_2013_15 == 0 & sickpay_2013_15_1BA == 0 & disabled_2019 == 0

//2020

tabulate woman if desc == 1 & reg_stat_2020 == '1' & firm_emp_2017_19 == 1 & inrange(age_2019, 30, 62) & WAA_2014_16 == 1 & disabled_2020 == 0

tabulate woman if desc == 1 & reg_stat_2020 == '1' & firm_emp_2017_19 == 1 & inrange(age_2019, 30, 62) & sickpay_2014_16_1BA == 1 & disabled_2020 == 0

tabulate woman if desc == 1 & reg_stat_2020 == '1' & firm_emp_2017_19 == 1 & inrange(age_2019, 30, 62) & WAA_2014_16 == 0 & sickpay_2014_16_1BA == 0 & disabled_2020 == 0

// EXAMINE INCOME DIFFERENTIALS, BY HEALTH STATUS

// step one: entire analysis sample

// pre crisis

summarize work_income_2019 if reg_stat_2019 == '1' & firm_emp_2017_19 == 1 & inrange(age_2019, 30, 62) & WAA_2014_16 == 1 & disabled_2020 == 0

summarize work_income_2019 if reg_stat_2019 == '1' & firm_emp_2017_19 == 1 & inrange(age_2019, 30, 62) & sickpay_2014_16_1BA == 1 & disabled_2020 == 0

summarize work_income_2019 if reg_stat_2019 == '1' & firm_emp_2017_19 == 1 & inrange(age_2019, 30, 62) & WAA_2014_16 == 0 & sickpay_2014_16_1BA == 0 & disabled_2020 == 0

// post crisis

summarize work_income_2021 if reg_stat_2021 == '1' & firm_emp_2017_19 == 1 & inrange(age_2019, 30, 62) & WAA_2014_16 == 1 & disabled_2020 == 0

summarize work_income_2021 if reg_stat_2021 == '1' & firm_emp_2017_19 == 1 & inrange(age_2019, 30, 62) & sickpay_2014_16_1BA == 1 & disabled_2020 == 0

summarize work_income_2021 if reg_stat_2021 == '1' & firm_emp_2017_19 == 1 & inrange(age_2019, 30, 62) & WAA_2014_16 == 0 & sickpay_2014_16_1BA == 0 & disabled_2020 == 0

// step two: only those exposed to unemployment during 2020

// pre crisis

summarize work_income_2019 if reg_stat_2019 == '1' & firm_emp_2017_19 == 1 & inrange(age_2019, 30, 62) & WAA_2014_16 == 1 & disabled_2020 == 0 & unemp_2020 == 1

summarize work_income_2019 if reg_stat_2019 == '1' & firm_emp_2017_19 == 1 & inrange(age_2019, 30, 62) & sickpay_2014_16_1BA == 1 & disabled_2020 == 0 & unemp_2020 == 1

summarize work_income_2019 if reg_stat_2019 == '1' & firm_emp_2017_19 == 1 & inrange(age_2019, 30, 62) & WAA_2014_16 == 0 & sickpay_2014_16_1BA == 0 & disabled_2020 == 0 & unemp_2020 == 1

// post crisis

summarize work_income_2021 if reg_stat_2021 == '1' & firm_emp_2017_19 == 1 & inrange(age_2019, 30, 62) & WAA_2014_16 == 1 & disabled_2020 == 0 & unemp_2020 == 1

summarize work_income_2021 if reg_stat_2021 == '1' & firm_emp_2017_19 == 1 & inrange(age_2019, 30, 62) & sickpay_2014_16_1BA == 1 & disabled_2020 == 0 & unemp_2020 == 1

summarize work_income_2021 if reg_stat_2021 == '1' & firm_emp_2017_19 == 1 & inrange(age_2019, 30, 62) & WAA_2014_16 == 0 & sickpay_2014_16_1BA == 0 & disabled_2020 == 0 & unemp_2020 == 1

// EXAMINE LABOR MARKET MOBILITY 2021

summarize unemp_2021 unemp_median_2021 BA_3_5_2021 if reg_stat_2021 == '1' & firm_emp_2017_19 == 1 & inrange(age_2019, 30, 62) & WAA_2014_16 == 1 & disabled_2020 == 0

summarize unemp_2021 unemp_median_2021 BA_3_5_2021 if reg_stat_2021 == '1' & firm_emp_2017_19 == 1 & inrange(age_2019, 30, 62) & sickpay_2014_16_1BA == 1 & disabled_2020 == 0

summarize unemp_2021 unemp_median_2021 BA_3_5_2021 if reg_stat_2021 == '1' & firm_emp_2017_19 == 1 & inrange(age_2019, 30, 62) & WAA_2014_16 == 0 & sickpay_2014_16_1BA == 0 & disabled_2020 == 0

summarize unemp_2021 unemp_median_2021 BA_3_5_2021 if reg_stat_2021 == '1' & firm_emp_2017_19 == 1 & inrange(age_2019, 30, 62) & WAA_2014_16 == 1 & disabled_2020 == 0 & unemp_2020 == 1

summarize unemp_2021 unemp_median_2021 BA_3_5_2021 if reg_stat_2021 == '1' & firm_emp_2017_19 == 1 & inrange(age_2019, 30, 62) & sickpay_2014_16_1BA == 1 & disabled_2020 == 0 & unemp_2020 == 1

summarize unemp_2021 unemp_median_2021 BA_3_5_2021 if reg_stat_2021 == '1' & firm_emp_2017_19 == 1 & inrange(age_2019, 30, 62) & WAA_2014_16 == 0 & sickpay_2014_16_1BA == 0 & disabled_2020 == 0 & unemp_2020 == 1

// EXAMINE COMPOSITIONAL DIFFS AMONG UNEMP BEN RECIPIENTS

summarize WAA_2013_15 sickpay_2013_15_1BA age_2018 woman married_2018 educ_high_2018 educ_medium_2018 educ_low_2018 immi desc if reg_stat_2019 == '1' & firm_emp_2016_18 == 1 & inrange(age_2018, 30, 62) & disabled_2019 == 0 & unemp_2019 == 1

summarize WAA_2014_16 sickpay_2014_16_1BA age_2019 woman married_2019 educ_high_2019 educ_medium_2019 educ_low_2019 immi desc if reg_stat_2020 == '1' & firm_emp_2017_19 == 1 & inrange(age_2019, 30, 62) & disabled_2020 == 0 & unemp_2020 == 1

// sum stats for descendants

tabulate woman if desc == 1 & reg_stat_2019 == '1' & firm_emp_2016_18 == 1 & inrange(age_2018, 30, 62) & WAA_2013_15 == 1 & disabled_2019 == 0 & unemp_2019 == 1

tabulate woman if desc == 1 & reg_stat_2020 == '1' & firm_emp_2017_19 == 1 & inrange(age_2019, 30, 62) & WAA_2014_16 == 1 & disabled_2020 == 0 & unemp_2020 == 1

// correlation matrix

correlate unemp_2019 WAA_2013_15 sickpay_2013_15_1BA age_2018 woman married_2018 educ_high_2018 educ_medium_2018 educ_low_2018 immi desc if reg_stat_2019 == '1' & firm_emp_2016_18 == 1 & inrange(age_2018, 30, 62) & disabled_2019 == 0

correlate unemp_2020 WAA_2014_16 sickpay_2014_16_1BA age_2019 woman married_2019 educ_high_2019 educ_medium_2019 educ_low_2019 immi desc if reg_stat_2020 == '1' & firm_emp_2017_19 == 1 & inrange(age_2019, 30, 62) & disabled_2020 == 0

/// RUNNING REGRESSIONS

// 2019

regress unemp_2019 WAA_2013_15 sickpay_2013_15_1BA if reg_stat_2019 == '1' & firm_emp_2016_18 == 1 & inrange(age_2018, 30, 62) & disabled_2019 == 0

regress unemp_2019 WAA_2013_15 sickpay_2013_15_1BA age_2018 age2_2018 woman married_2018 immi desc educ_low_2018 educ_medium_2018 if reg_stat_2019 == '1' & firm_emp_2016_18 == 1 & inrange(age_2018, 30, 62) & disabled_2019 == 0

regress unemp_2019 WAA_2013_15 sickpay_2013_15_1BA age_2018 age2_2018 woman married_2018 immi desc educ_low_2018 educ_medium_2018 WAA_low_edu_1315 WAA_med_edu_1315 WAA_immi_1315 WAA_desc_1315 sickp_low_edu_1315 sickp_med_edu_1315 sickp_immi_1315 sickp_desc_1416 if reg_stat_2019 == '1' & firm_emp_2016_18 == 1 & inrange(age_2018, 30, 62) & disabled_2019 == 0

// 2020

regress unemp_2020 WAA_2014_16 sickpay_2014_16_1BA if reg_stat_2020 == '1' & firm_emp_2017_19 == 1 & inrange(age_2019, 30, 62) & disabled_2020 == 0

regress unemp_2020 WAA_2014_16 sickpay_2014_16_1BA age_2019 age2_2019 woman married_2019 immi desc educ_low_2019 educ_medium_2019 if reg_stat_2020 == '1' & firm_emp_2017_19 == 1 & inrange(age_2019, 30, 62) & disabled_2020 == 0

regress unemp_2020 WAA_2014_16 sickpay_2014_16_1BA age_2019 age2_2019 woman married_2019 immi desc educ_low_2019 educ_medium_2019 WAA_low_edu_1416 WAA_med_edu_1416 WAA_immi_1416 WAA_desc_1416 sickp_low_edu_1416 sickp_med_edu_1416 sickp_immi_1416 sickp_desc_1416 if reg_stat_2020 == '1' & firm_emp_2017_19 == 1 & inrange(age_2019, 30, 62) & disabled_2020 == 0

/// ROBUSTNESS CHECKS

// LOGIT MODEL

// 2019

logit unemp_2019 WAA_2013_15 sickpay_2013_15_1BA if reg_stat_2019 == '1' & firm_emp_2016_18 == 1 & inrange(age_2018, 30, 62) & disabled_2019 == 0, or

logit unemp_2019 WAA_2013_15 sickpay_2013_15_1BA age_2018 age2_2018 woman married_2018 immi desc educ_low_2018 educ_medium_2018 if reg_stat_2019 == '1' & firm_emp_2016_18 == 1 & inrange(age_2018, 30, 62) & disabled_2019 == 0, or

// 2020

logit unemp_2020 WAA_2014_16 sickpay_2014_16_1BA if reg_stat_2020 == '1' & firm_emp_2017_19 == 1 & inrange(age_2019, 30, 62) & disabled_2020 == 0, or

logit unemp_2020 WAA_2014_16 sickpay_2014_16_1BA age_2019 age2_2019 woman married_2019 immi desc educ_low_2019 educ_medium_2019 if reg_stat_2020 == '1' & firm_emp_2017_19 == 1 & inrange(age_2019, 30, 62) & disabled_2020 == 0, or

// FIRMER LM ATTACHMENT

// 2019

regress unemp_2019 WAA_2013_15 sickpay_2013_15_1BA if reg_stat_2019 == '1' & firm_emp_min2Y_2016_18 == 1 & inrange(age_2018, 30, 62) & disabled_2019 == 0

regress unemp_2019 WAA_2013_15 sickpay_2013_15_1BA age_2018 age2_2018 woman married_2018 immi desc educ_low_2018 educ_medium_2018 if reg_stat_2019 == '1' & firm_emp_min2Y_2016_18 == 1 & inrange(age_2018, 30, 62) & disabled_2019 == 0

// 2020

regress unemp_2020 WAA_2014_16 sickpay_2014_16_1BA if reg_stat_2020 == '1' & firm_emp_min2Y_2017_19 == 1 & inrange(age_2019, 30, 62) & disabled_2020 == 0

regress unemp_2020 WAA_2014_16 sickpay_2014_16_1BA age_2019 age2_2019 woman married_2019 immi desc educ_low_2019 educ_medium_2019 if reg_stat_2020 == '1' & firm_emp_min2Y_2017_19 == 1 & inrange(age_2019, 30, 62) & disabled_2020 == 0

// EXCLUDING SHORT-TERM UNEMP

// 2019

regress unemp_median_2019 WAA_2013_15 sickpay_2013_15_1BA if reg_stat_2019 == '1' & firm_emp_2016_18 == 1 & inrange(age_2018, 30, 62) & disabled_2019 == 0

regress unemp_median_2019 WAA_2013_15 sickpay_2013_15_1BA age_2018 age2_2018 woman married_2018 immi desc educ_low_2018 educ_medium_2018 if reg_stat_2019 == '1' & firm_emp_2016_18 == 1 & inrange(age_2018, 30, 62) & disabled_2019 == 0

// 2020

regress unemp_median_2020 WAA_2014_16 sickpay_2014_16_1BA if reg_stat_2020 == '1' & firm_emp_2017_19 == 1 & inrange(age_2019, 30, 62) & disabled_2020 == 0

regress unemp_median_2020 WAA_2014_16 sickpay_2014_16_1BA age_2019 age2_2019 woman married_2019 immi desc educ_low_2019 educ_medium_2019 if reg_stat_2020 == '1' & firm_emp_2017_19 == 1 & inrange(age_2019, 30, 62) & disabled_2020 == 0

// EXCLUDING LONG-TERM UNEMP

// 2019

regress unemp_max1BA_2019 WAA_2013_15 sickpay_2013_15_1BA if reg_stat_2019 == '1' & firm_emp_2016_18 == 1 & inrange(age_2018, 30, 62) & disabled_2019 == 0

regress unemp_max1BA_2019 WAA_2013_15 sickpay_2013_15_1BA age_2018 age2_2018 woman married_2018 immi desc educ_low_2018 educ_medium_2018 if reg_stat_2019 == '1' & firm_emp_2016_18 == 1 & inrange(age_2018, 30, 62) & disabled_2019 == 0

// 2020

regress unemp_max1BA_2020 WAA_2014_16 sickpay_2014_16_1BA if reg_stat_2020 == '1' & firm_emp_2017_19 == 1 & inrange(age_2019, 30, 62) & disabled_2020 == 0

regress unemp_max1BA_2020 WAA_2014_16 sickpay_2014_16_1BA age_2019 age2_2019 woman married_2019 immi desc educ_low_2019 educ_medium_2019 if reg_stat_2020 == '1' & firm_emp_2017_19 == 1 & inrange(age_2019, 30, 62) & disabled_2020 == 0

// WORSE HEALTH

// 2019

regress unemp_2019 WAA_min2Y_2013_15 sickpay_min2Y_2013_15_1BA if reg_stat_2019 == '1' & firm_emp_2016_18 == 1 & inrange(age_2018, 30, 62) & disabled_2019 == 0

regress unemp_2019 WAA_min2Y_2013_15 sickpay_min2Y_2013_15_1BA age_2018 age2_2018 woman married_2018 immi desc educ_low_2018 educ_medium_2018 if reg_stat_2019 == '1' & firm_emp_2016_18 == 1 & inrange(age_2018, 30, 62) & disabled_2019 == 0

// 2020

regress unemp_2020 WAA_min2Y_2014_16 sickpay_min2Y_2014_16_1BA if reg_stat_2020 == '1' & firm_emp_2017_19 == 1 & inrange(age_2019, 30, 62) & disabled_2020 == 0

regress unemp_2020 WAA_min2Y_2014_16 sickpay_min2Y_2014_16_1BA age_2019 age2_2019 woman married_2019 immi desc educ_low_2019 educ_medium_2019 if reg_stat_2020 == '1' & firm_emp_2017_19 == 1 & inrange(age_2019, 30, 62) & disabled_2020 == 0

// GENDER SPLIT

// 2019

regress unemp_2019 WAA_2013_15 sickpay_2013_15_1BA if reg_stat_2019 == '1' & firm_emp_2016_18 == 1 & inrange(age_2018, 30, 62) & woman ==0 & disabled_2019 == 0

regress unemp_2019 WAA_2013_15 sickpay_2013_15_1BA age_2018 age2_2018 married_2018 immi desc educ_low_2018 educ_medium_2018 if reg_stat_2019 == '1' & firm_emp_2016_18 == 1 & inrange(age_2018, 30, 62) & woman ==0 & disabled_2019 == 0

regress unemp_2019 WAA_2013_15 sickpay_2013_15_1BA if reg_stat_2019 == '1' & firm_emp_2016_18 == 1 & inrange(age_2018, 30, 62) & woman ==1 & disabled_2019 == 0

regress unemp_2019 WAA_2013_15 sickpay_2013_15_1BA age_2018 age2_2018 married_2018 immi desc educ_low_2018 educ_medium_2018 if reg_stat_2019 == '1' & firm_emp_2016_18 == 1 & inrange(age_2018, 30, 62) & woman ==1 & disabled_2019 == 0

// 2020

regress unemp_2020 WAA_2014_16 sickpay_2014_16_1BA if reg_stat_2020 == '1' & firm_emp_2017_19 == 1 & inrange(age_2019, 30, 62) & woman ==0 & disabled_2020 == 0

regress unemp_2020 WAA_2014_16 sickpay_2014_16_1BA age_2019 age2_2019 married_2019 immi desc educ_low_2019 educ_medium_2019 if reg_stat_2020 == '1' & firm_emp_2017_19 == 1 & inrange(age_2019, 30, 62) & woman ==0 & disabled_2020 == 0

regress unemp_2020 WAA_2014_16 sickpay_2014_16_1BA if reg_stat_2020 == '1' & firm_emp_2017_19 == 1 & inrange(age_2019, 30, 62) & woman ==1 & disabled_2020 == 0

regress unemp_2020 WAA_2014_16 sickpay_2014_16_1BA age_2019 age2_2019 married_2019 immi desc educ_low_2019 educ_medium_2019 if reg_stat_2020 == '1' & firm_emp_2017_19 == 1 & inrange(age_2019, 30, 62) & woman ==1 & disabled_2020 == 0
